# A systematic review of the clustering and correlates of physical activity and sedentary behavior among boys and girls

**DOI:** 10.1186/s12889-022-14869-0

**Published:** 2023-02-21

**Authors:** Gabrielli Thais de Mello, Cecília Bertuol, Giseli Minatto, Valter Cordeiro Barbosa Filho, Brian Oldenburg, Rebecca Maree Leech, Kelly Samara Silva

**Affiliations:** 1 Research Center for Physical Activity and Health, School of Sports, Campus João David Ferreira Lima, Federal University of Santa Catarina, Florianópolis, Room 48, Florianópolis, SC 88040-900 Brazil; 2 Federal Institute of Education, Science and Technology of Ceara, Aracati, Brazil; 3 Implementation Science Lab, Baker Heart and Diabetes Institute, Melbourne, 3004 Australia; 4 School of Psychology and Public Health, La Trobe University, Melbourne, 3086 Australia; 5 Institute for Physical Activity and Nutrition (IPAN), Deakin University, Geelong, Victoria Australia

**Keywords:** Cluster analysis, Adolescent, Children

## Abstract

Identifying the clustering and correlates of physical activity (PA) and sedentary behavior (SB) is very important for developing appropriate lifestyle interventions for children and adolescents. This systematic review (Prospero CRD42018094826) aimed to identify PA and SB cluster patterns and their correlates among boys and girls (0–19 years). The search was carried out in five electronic databases. Cluster characteristics were extracted in accordance with authors’ descriptions by two independent reviewers and a third resolved any disagreements. Seventeen studies met the inclusion criteria and the population age ranged from six to 18 years old. Nine, twelve, and ten cluster types were identified for mixed-sex samples, boys, and girls, respectively. While girls were in clusters characterized by “Low PA Low SB” and “Low PA High SB”, the majority of boys were in clusters defined by “High PA High SB” and “High PA Low SB”. Few associations were found between sociodemographic variables and all cluster types. Boys and girls in “High PA High SB” clusters had higher BMI and obesity in most of the tested associations. In contrast, those in the “High PA Low SB” clusters presented lower BMI, waist circumference, and overweight and obesity. Different cluster patterns of PA and SB were observed in boys and girls. However, in both sexes, a better adiposity profile was found among children and adolescents in “High PA Low SB” clusters. Our results suggest that it is not enough to increase PA to manage the adiposity correlates, it is also necessary to reduce SB in this population.

**Supplementary Information:**

The online version contains supplementary material available at 10.1186/s12889-022-14869-0.

## Introduction

Clustering among physical activity (PA) and sedentary behavior (SB) have been linked to important health outcomes (e.g. cardio-metabolic biomarkers, adiposity, self-esteem and psychological distress) [1–4]. PA and SB are coexisting behaviors and form part of the human movement spectrum [5]. Thus, an increase in PA may not be associated with a decrease in SB and vice versa, suggesting that this behavioral pattern coexists in different ways [6–8].

Recent studies have shown that low levels of PA combined with excessive time spent in SB occur repeatedly in children and adolescents [9–11]. Previous reviews have noted that clusters characterized by “High levels of PA and High time in SB” [8], “High PA and Low SB” and “Low PA and High SB” [6, 8] occurred most frequently in children and adolescents. Additionally, one review has identified a tendency for older children/adolescents to comprise clusters defined by low PA [12]. Considering characteristics of the clusters, in relation to sex, girls tend to be in clusters characterized by low PA and high time spent in socializing activities, whereas boys tend be in clusters characterized by high PA and high time spent watching television and playing videogame [13–18]. These findings suggest that both age and sex are important factors to consider when examining PA and SB cluster patterns. This is further supported by evidence showing the prevalence of compliance with PA and SB guidelines decreases and increases with increasing age, respectively [19, 20] and the widening of differences in PA levels and time spent in SB between boys and girls between childhood and adolescence [21].

These clusters with distinct characteristics may also correspond to correlates in different ways. Thus, the association between clusters and different sociodemographic, mental and physical health have been explored in children and adolescents [1, 12, 14, 18]. Studies suggest that better cardiometabolic health, self-esteem, body image and weight status are found in youth with the healthiest behavioral clusters [1, 22, 23]. For example, adolescents in “uses recreation center” and “active in school” clusters had higher self-esteem [23]. The opposite has also been observed for children and adolescents in less healthy cluster. For example, boys and girls in clusters characterized by “low PA and SB” and “high PA and SB” higher adiposity levels adiposity [24–26].

Given the complex inter-relationships summarized above, there is a need to (i) map the characteristics of PA and SB cluster patterns among boys and girls according to the methodological quality of studies; (ii) describe which clusters are most prevalent by sex; and (iii) examine the range of correlates that have been explored. This is necessary because previous reviews on cluster patterns were either not systematic [12], employed limited search strategies (i.e., limited combination of descriptors for PA and SB) [6, 12, 14] and/or limited the publications reviewed up to 2018 [6]. To identify different patterns and their correlates will help to inform the development of appropriate strategies for modifying and improving the lifestyles of different population subgroups [27–29].

The aim of the present study is therefore to review systematically the literature that has investigated the clustering patterns of PA and SB in children and adolescents. In particular, we aimed to verify if clusters differ according to sex, and to identify their potential correlates.

## Methods

### Protocol

This systematic review used Preferred Reporting Items for Systematic Reviews and Meta-Analysis (PRISMA) [30, 31] and the extension Synthesis Without Meta-analysis (SWiM) [32]. PRISMA and SWiM checklist is included in Supplementary material (Table S1 and Table S2). This study was registered in PROSPERO (CRD42018094826) and formed part of a comprehensive evidence synthesis project [8]. The PI(E)COS strategy was used for the development of the research question.

### Eligibility criteria

Studies were included if they met the following eligibility criteria: (a) included children and/or adolescents (aged 0–19 years, or reported means between these ages); (b) analyzed simultaneously PA and SB); c) applied exploratory data-based statistical procedures, considering cluster analysis (i.e., k-means), latent Class/Profile Analysis, and dimensionality reduction techniques (i.e., Principal Component Analysis and Factor Analysis); and (d) be published in English, Portuguese, or Spanish. All correlates reported in the included studies were extracted. Studies were excluded if they involved clinical populations (e.g., disabilities, metabolic and/or cardiovascular diseases, hospitalized or institutionalized populations), or included other behaviors or variables (e.g., tobacco use, unhealthy eating, socioeconomic status) as part of the cluster patterns. Reviews, letters to editor, and conference abstracts were excluded. All studies designs were considered for inclusion. More information about the eligibility criteria can be observed in Supplementary material Table S3.

### Search strategies and selection process

The search strategies used five electronic databases (PubMed, Scopus, Web of Science, LILACS and PsycINFO) and were carried out in December 2019. Particularities strategy and Boolean operators and truncation symbols ($, * or "") were considered and no restrictions of publication year and study design were applied. The search string can be observed in Supplementary material (Table S4).

**Fig. 1:**
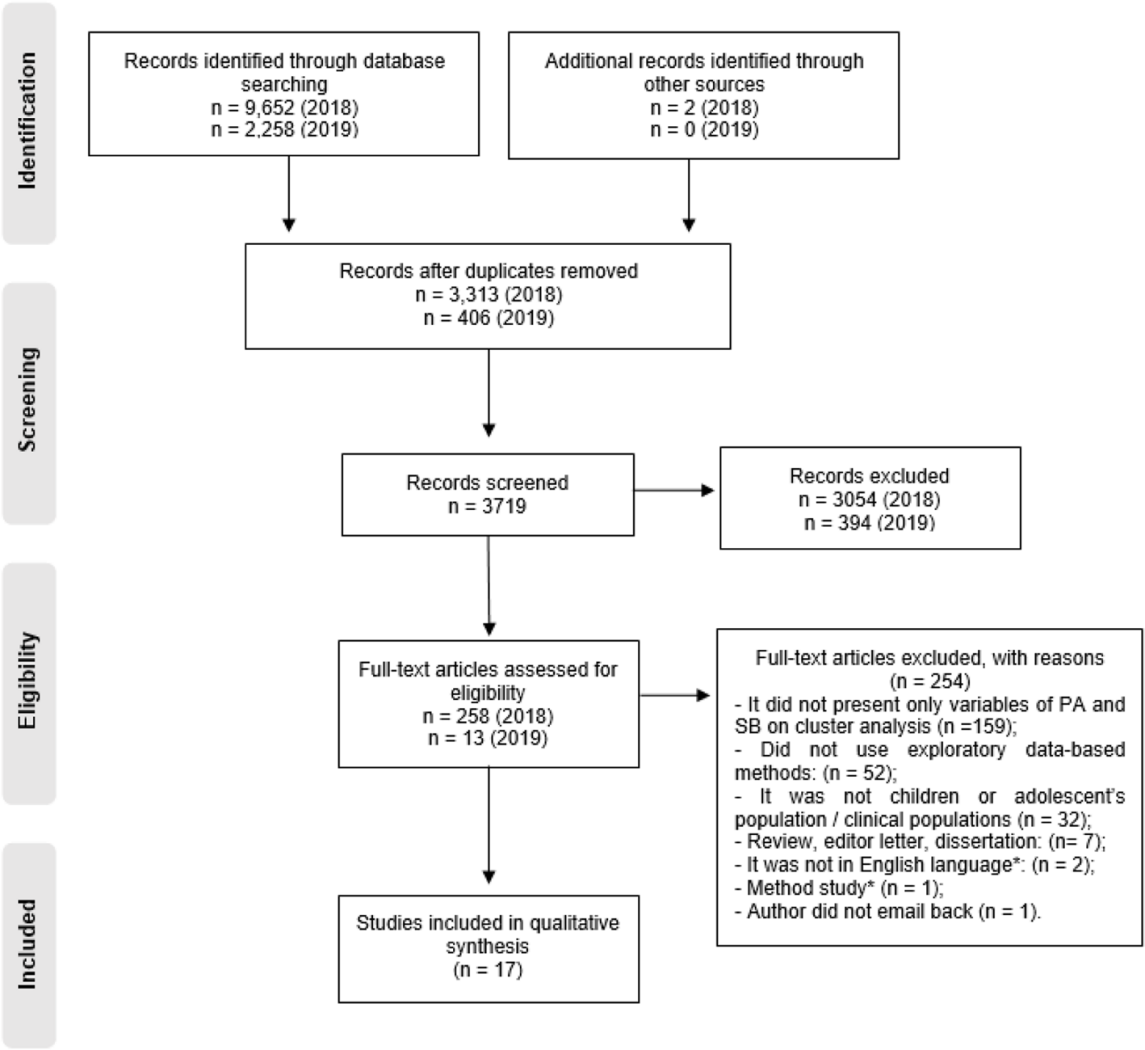
Flow of study inclusion for the review. Note: * Polash idiom; Explained how to use cluster analysis – did not present results

Firstly, the titles and abstracts were screened independently by the authors of the first review (GTM/RMC and GTM/MVVL). If the relevance of an article was unclear, it was retained for full text screening by the same peers. Reference lists of included studies and previous reviews were examined as additional searches (RMC and MVVL). More information can be observed in Fig. 1.

### Methodological quality assessment of included studies

The methodological quality of the included studies was assessed by the 17-point adapted version of the Quality Assessment Tool for Quantitative Studies of Effective Public Health Practice Project (EPHPP) [33], in four methodological domains, as shown in supplementary material Table S5. The risk of bias classification (low [strong], moderate [moderate] and high [weak]) for each domain was determined on the basis of the study distribution (see Table S6 supplementary material). The risk of bias was assessed by two independent reviewer (GTM and GM) and a third reviewer was consulted for the consensus of disagreements (CB).

### Data extraction and synthesis

Data were extracted by (GTM/CB) and discrepancies were resolved by a third person (GM). Extraction elements included: (1) article description (e.g., publication year; country; study design; sample size and age); (2) instruments used to measure PA and SB; behaviors domain and components (e.g., leisure-time PA, habitual PA, daily time spent on TV, videogames); (3) variables used to determine clusters (i.e. cluster input variables) and the resulting cluster types according to mixed-sex samples, boys, girls, children, and adolescents; and (4) all correlates examined and their direction of association.

Instruments used to measure PA and SB were classified as: (1) Defined (with validation process); (2) Undefined (reported question and/or response option and instrument reference); (3) Undefined-Reproducible (reported question and response options but did not mention the reference); (4) Objective measurement (e.g., accelerometer); (see Table 2 and Figure S1a and S1b in supplementary material).

The descriptions reported by the authors of the studies were used to extract cluster characteristics according to mixed-sex, boys and girls. For example, authors characterized a cluster with low values for watching TV and high values for playing games and low PA levels; the cluster type was classified as “Low PA and High/Low SB”. Where authors did not provide a text description, quantitative data presented in figures and/or tables were used to classify cluster types. Thus, labels of PA and SB components were categorized as “Low” or “High”.

Paper characteristics included in this review were described in the light of the total number of studies, thus, articles reporting on the same data set were represented by the most recently published paper. All other sections of the results were described taking into account the total number of articles included in the review. For the cluster descriptions, similar clusters derived from the same population, and presented in different articles, were therefore reported only once. A meta-analysis was not performed due to the heterogeneity observed between studies in the following aspects 1) Distinctions in measurements and indicator types of PA and SB; 2) Variability of algorithms used in distinct data-based cluster statistical procedures; and, 3) The different clusters types identified.

**Fig. 2:**
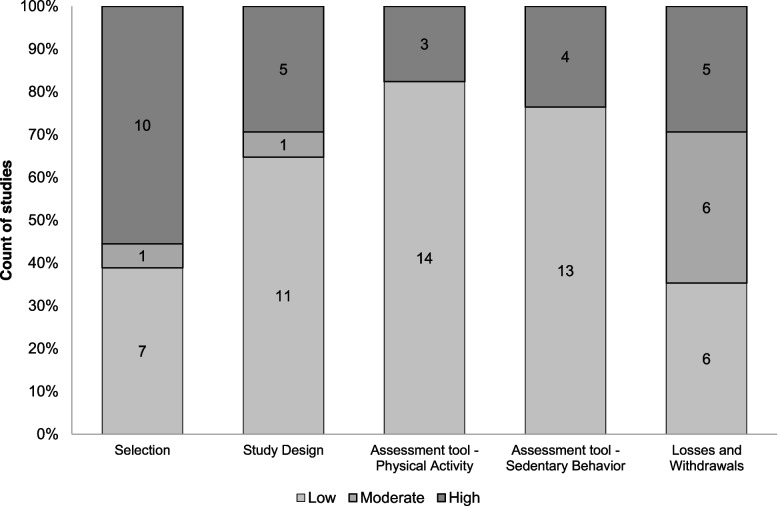
Assessment of the risk of bias of studies

The results were organized according to the SWiM as follow: a) study characteristics and its risk of bias (Table 1 and Fig. 2); b) instruments used to evaluated PA and SB, and variables used in clusters procedures (Table 2 and Table 3); c) cluster types identified and their correlates (Table 4 and 5). Excel was used to make the figures and tables. Correlates were categorized as sociodemographic, adiposity, healthy risk behaviors and others.

**Table 1:** Characteristics of studies included in the systematic review (*n* = 17)

First author (publication year)	Country	Original project	Sample size (girls %)	Age group (mean age)	Method used to derive clusters	Number of clusters	Correlates associated with clusters
De Bourdeaudhuij (2013) [34]	European countries^**a**^	ENERGY	766 (52.9%)	10 – 12 years (11.5 girls /11.7 boys)	Two step cluster analysis (hierarchical and non-hierarchical methods)	Boys 4 Girls 4	BMI and waist circumference
Gorely (2007) [15]	UK^**b**^	STIL	1,371 (62.0%)	Mean 14.7 years (sd = 0.92, range 12.5–17.6 years)	Cluster analysis (Ward’s method and k-means)	Boys 5 Girls 5	None
Huang (2016) [17]	China^**c**^	Not reported	951 (50.5%)	9 – 13 years (11.0)	Cluster analysis (hierarchical)	Boys 5 Girls 5	Sociodemographic factors and sports team participation
Kim (2016) [26]	USA	YRBS	12,081 (49.4%)	9th – 12th grades (adolescents)	Latent class analysis	Boys 4 Girls 4	Obesity
Lazarou (2009) [35]	Cyprus	CYKIDS	1,140 (53.4%)	10 – 13 years (10.7)	Principal component analysis	8	None
Marshall (2002) [36]	USA and UK	Not reported	USA: 1,750 (59.0%) UK: 744 (85.0%)	USA: mean 12.9 years (sd = 0.92) UK: mean 13.0 years (sd = 0.94)	Cluster analysis	Boys 3 Girls 3	Age, nationality, ethnicity, and BMI
Melkevik (2011) [25]	Norway	HBSC	4,848 (48.0%)	13, 15, and 16 years	Latent profile analysis	Boys 6 Girls 6	Overweight
Nelson (2005) [37]	USA	Add Health	1,1957 (50.0%)	Mean age (wave I) 14.9 years (sd = 0.12)	Cluster analysis*	7	Meet PA guidelines
Nelson (2006) [23]	USA	Add Health	1,1957 (50.0%)	Mean age (wave II) 15.8 years (sd = 11.6)	Cluster analysis*	7	Health risk behaviors and other weekly activities, and self-esteem
O'Neill (2017) [38]	Ireland	GUI	8,568 (48.9%)	9 – 13 years	Two step cluster analysis	Boys 4 Girls (no coherent cluster type found)	Weight status
Patnode (2011) [24]	USA	IDEA and ECHO	720 (51.1%)	Mean age 14.7 years (sd = 1.8)	Latent class analysis	Boys 3 Girls 3	Grade, race, parent education, live with 2 parents, overweight, weight status, free or reduced-price lunch
Ramos (2012) [39]	Spain	HBSC	21,811 (53.1%)	11 – 18 years	Cluster analysis (general linear models)	3	Biopsychosocial health
Spengler (2015) [16]	Germany	MoMo	2,083 (50.5%)	11 – 13 years 14 – 17 years	Cluster K-means	Boys 8 Girls 7	Age and socioeconomic status
Taverno Ross (2016) [40]	USA	TRACK	495 (55.4%)	5th (baseline) and 7th grades (children)	Latent class analysis	Boys 3 Girls 3	Socio-demographics, Individual-level factors and Interpersonal-level factors; School-level factors^#^
te Velde (2007) [13]	European countries^**d**^	CSS	12,538 (50.1%)	8.8 – 13.8 years (11.4)	Cluster K-means	Boys 5 Girls 5	Overweight
Wang (2006) [41]	Singapore	Not reported	780 (61.8%)	11 – 14 years	Cluster analysis (hierarchical methods)	Boys 3 Girls 3	None
Wang (2012) [42]	Singapore	Not reported	847 (61.0%)	10 – 16 years	Latent profile analysis	5	None

*USA* United States, *UK* United Kingdom, *ENERGY* European energy balance research to prevent excessive weight Gain among youth, *NHANES* National health and nutrition examination survey, *STIL* Project sedentary teenagers and inactive lifestyles, *YRBS* Youth risk behavior survey, *CYKIDS* Cyprus kids study, *HBSC* Health behavior in school-aged children, *Add Health* National longitudinal study of adolescent health, *GUI* Growing up in Ireland, *IDEA* Eating and activity in adolescents, *ECHO* Etiology of childhood obesity, *MoMo* Motorik-modul study, *TRACK* Transitions and activity changes in kids, *BMI* Body mass index, *BMI* Body mass index

^a^ Belgium, Greece, Hungary, the Netherlands, and Switzerland

^b^ England, Northern Ireland, Scotland, and Wales

^c^ Hong Kong Island, Kowloon, and the New Territories in Hong Kong

^d^ Austria, Belgium, Denmark, Iceland, the Netherlands, Norway, Portugal, Spain, and Sweden

^*^Did not specify which cluster analysis

^#^ race, Parent education, SES, Weight status, Self-efficacy, enjoyment, Perceived PA barriers, Perceived parent support for PA, Parent support for PA, Sports/physically active lessons in past year, Screen devices in bedroom, Home PA equipment, Neighborhood safety

**Table 2:** Classification of instruments used to measure PA and SB

Author (publication year)	Instruments classification	
	**Physical Activity**	**Sedentary Behavior**
De Bourdeaudhuij (2013) [34]	Accelerometer (Defined)	Accelerometer (Defined)
Gorely (2007) [15]	Defined	Defined
Huang (2016) [17]	Validated	Validated
Kim (2016) [26]	Undefined-Reproducible	Undefined-Reproducible
Lazarou (2009) [35]	Undefined-Reproducible	Undefined-Reproducible
Marshall (2002) [36]	Defined	Defined
Melkevik (2011) [25]	Defined	Defined
Nelson (2005) [37]	Defined	Undefined
Nelson (2006) [23]	Defined	Undefined
O'Neill (2017) [38]	Undefined-Reproducible	Undefined-Reproducible
Patnode (2011) [24]	Accelerometer (Defined)*	Defined
Ramos (2012) [39]	Defined	Defined
Spengler (2015) [16]	Defined	Defined
Taverno Ross (2016) [40]	Accelerometer (Defined)*	Defined
Te velde (2007) [13]	Defined	Defined
Wang (2006) [41]	Defined	Defined
Wang (2011) [42]	Defined	Defined

^*^ Used two instruments (accelerometer and questionnaire). (1) Defined (reported the validation process); (2) Undefined (reported question and/or response option and instrument reference); (3) Undefined-Reproducible (reported question and response options but no instrument reference); (4) Objective measurement (e.g., accelerometer)

**Table 3:**
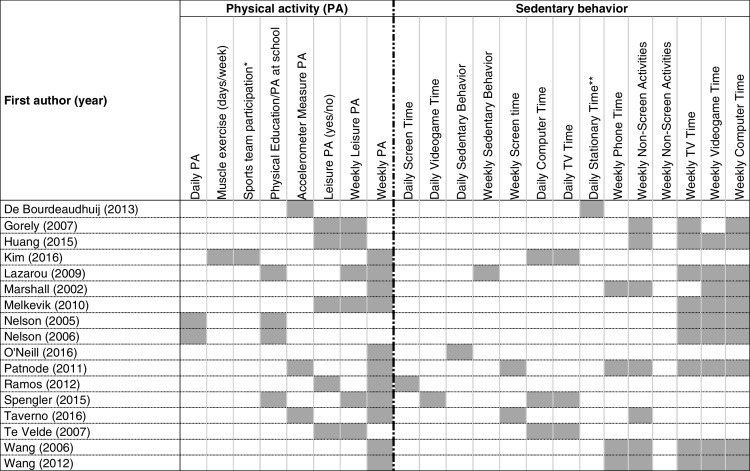
The PA and SB variables used to determine the behavioral clusters in each study

*Number of modalities; ** Note: *Stationary time refers to accelerometer measured movement behaviors

**Table 4:**
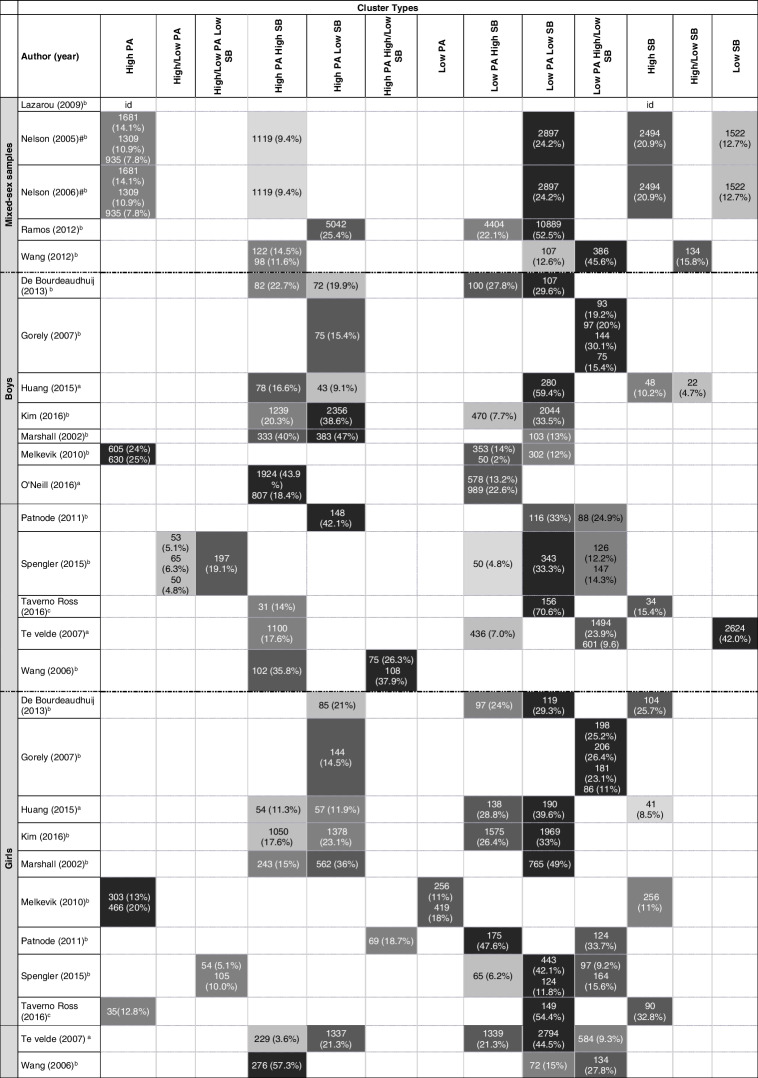
Description of the derived clusters and the prevalence of children and adolescents within each cluster. Results are presented as n(%)

*PA* Physical activity, *SB* Sedentary behavior

#Same cluster. Id: Impossible to identify. In each column, the darker the gray, the greater number of children and adolescents in each cluster type. N and prevalence should be interpreted according to n sample present in each study (line of each study). More than one prevalence included in a little square means that more than one cluster were identified with this characteristic

^a^involved children and adolescent

^b^involved adolescents and average adolescents’ age

^c^involved children

**Table 5:** Summary of correlates examined and their associations with cluster types

Cluster Types													
**Correlates**	**High PA**	**High/Low PA**	**High/Low PA Low SB**	**High PA High SB**	**High PA Low SB**	**High PA High/Low SB**	**Low PA**	**Low PA High SB**	**Low PA Low SB**	**Low PA High/Low SB**	**High SB**	**High/Low SB**	**Low SB**
**Sociodemographic Factors**													
Age		0 ♂ [16] ≠ ♂ [16]	0 ♂ [16] ≠ ♂ [16] 0 ♀ [16] ≠ ♀ [16]	0 ♂ [17, 36] 0 ♀ [17] - ♀ older [36]	0 ♂ [17, 36] 0 ♀ [17] - ♀ older [36]			0 ♂ [16] ≠ ♂ [16] 0 ♀ [16, 17] ≠ ♀ [16]	0 ♂ [16, 17, 36] ≠ ♂ [16] 0 ♀ [17] + ♀ older [16, 36] ≠ ♀ [16]	0 ♂ [16] ≠ ♂ [16] 0 ♀ [16] ≠ ♀ [16]	0 ♂ [17] 0 ♀ [17]	0 ♂ [17]	
Race/Ethnic	0 ♀ [40]			0 ♂ [36, 40] 0 ♀ [36]	0 ♂ [24, 36] 0 ♀ [36]	0 ♀ [24]		- ♀ white [24]	0 ♂ [24, 36, 40] 0 ♀ [36, 40]	0 ♂ [24] + ♀ white [24]	0 ♂ [40] 0 ♀ [40]		
Parental Education	0 ♀ [40]			0 ♂ [17, 40] 0 ♀ [17]	0 ♂ [17, 24] 0 ♀ [17]	0 ♀ [24]		0 ♀ [17, 24]	0 ♂ [17, 24, 40] 0 ♀ [17, 40]	0 ♂ [24] 0 ♀ [24]	0 ♂ [40] - ♂ [17] 0 ♀ [17, 40]	0 ♂ [17]	
Socio economic status/poverty	0 ♀ [40]	0 ♂ [16]	0 ♂ [16] ≠ ♂ [16] 0 ♀ [16]	0 ♂ [40]				0 ♂ [16] ≠ ♂ [16] 0 ♀ [16] ≠ ♀ [16]	0 ♂ [16, 40] 0 ♀ [16, 40] ≠ ♀ [16]	0 ♂ [16] ≠ ♂ [16] 0 ♀ [16] ≠ ♀ [16]	0 ♂ [40] 0 ♀ [40]		
Live with 2 parents					0 ♂ [24]	+ ♀ [24]		- ♀ [24]	0 ♂ [24]	0 ♂ [24] + ♀ [24]			
Marital status				0 ♂ [17] 0 ♀ [17]	0 ♂ [17] 0 ♀ [17]			0 ♀ [17]	0 ♂ [17] 0 ♀ [17]		0 ♂ [17] 0 ♀ [17]	0 ♂ [17]	
Occupation				0 ♂ [17] 0 ♀ [17]	0 ♂ [17] 0 ♀ [17]			0 ♀ [17]	0 ♂ [17] 0 ♀ [17]		0 ♂ [17] 0 ♀ [17]	0 ♂ [17]	
Work	+ [23]			0 [23] + [23]					0 [23] + [23]				0 [23] + [23]
Nationality				0 ♂ [36] - ♀ North America [36]	0 ♂ [36] - ♀ North America [36]				0 ♂ [36] + ♀ North America [36]				
Grade					+ ♂ high 6th to 8th [24]	0 ♀ [24]		0 ♀ [24]	- ♂ less 6th to 8th [24]	- ♂ (less 6th to 8th) [24] 0 ♀ [24]			
Academic grades	+ [23]			0 [23]					0 [23]				0 [23]
Free or reduced-price lunch					0 ♂ [24]	0 ♀ [24]		0 ♀ [24]	0 ♂ [24]	0 ♂ [24] 0 ♀ [24]			
**Adiposity variables**													
BMI	0 ♀ [40]			0 ♂ [36, 40] 0 ♂ age 9 [38] - ♂ [34] + ♂ age 13 [38] 0 ♀ [36]	0 ♂ [36] - ♂ [34] 0 ♀ [36] - ♀ [34]			0 ♂ [34] + ♂ age 9 and 13 [38] 0 ♀ [34]	0 ♂ [34, 36, 40] 0 ♀ [34, 36, 40]		0 ♂ [40] 0 ♀ [34, 40]		
Waist circumference				- ♂ [34]	- ♂ [34] - ♀ [34]			0 ♂ [34] 0 ♀ [34]	0 ♂ [34] 0 ♀ [34] - ♀ [34]		0 ♀ [34] + ♀ [34]		
Weight status				0 ♂ [17, 40] 0 ♀ [17]	0 ♂ [17, 24] 0 ♀ [17]			0 ♀ [17]	0 ♂ [17, 24, 40] 0 ♀ [17]	0 ♂ [24]	0 ♂ [17, 40] 0 ♀ [17]	0 ♂ [17]	
Obesity				+ ♂ [26] + ♀ [26]				+ ♂ [26] + ♀ [26]	+ ♂ [26] + ♀ [26]				
Overweight	0 ♂ [25] 0 ♀ [25]			0 ♂ [13] 0 ♀ [13]	- ♂ [24]	- ♀ [24]	0 ♀ [25]	+ ♂ [13, 25] + ♀ [13, 24]	+ ♂ [24, 25] 0 ♀ [13]	+ ♂ [13, 24] - ♀ [24] + ♀ [13]	+ ♀ [25]		
Overweight + Obesity	0 ♀ [40]			0 ♂ [40]					0 ♂ [40] 0 ♀ [40]		0 ♂ [40] 0 ♀ [40]		
**Health risk behaviors**													
Delinquency	-[23]			0 [23]					-[23]				-[23]
Smoke	-[23]			-[23]					0 [23]				-[23]
Alcohol	-[23]			-[23]					+ [23]				-[23]
Drugs	-[23]			0 [23]					0 [23]				-[23]
Wear seatbelt	-[23]			-[23]					-[23]				-[23]
Sexual intercourse	-[23]			-[23]					0 [23]				-[23]
Truant	-[23]			-[23]					0 [23]				-[23]
**Others factors**													
Sleeps ≥ 8 h	+ [23]			+ [23]					-[23]				+ [23]
Siblings and sports team participation				0 ♂ [17] 0 ♀ [17]	0 ♂ [17] 0 ♀ [17]			0 ♀ [17]	0 ♂ [17] 0 ♀ [17]		0 ♂ [17] 0 ♀ [17]	0 ♂ [17]	
Meeting PA guidelines in adolescence	+ [37]			+ [37]					-[37]				+ [37]
Individual-level factors^a^	- self-steem [23] 0 ♀ self-eficacy [40]			- self-steem [23] + ♂ self-eficacy [40]					0 self-steem [23] - ♂ self-eficacy [40]0 ♀ self-eficacy [40]		- ♂ self-eficacy [40]0 ♀ self-eficacy [40]		0 self-steem [23]
Interpersonal-level factors^b^	0 ♀ [40]			0 ♂ [40]					0 ♂ [40] 0 ♀ [40]		0 ♂ [40] 0 ♀ [40]		
School-level factors^c^	0 ♀ [40]			0 ♂ [40]					0 ♂ [40] 0 ♀ [40]		0 ♂ [40] 0 ♀[40]		

*PA* Physical activity, *SB* Sedentary behavior. ♂ indicates male only. ♀ indicates female only. + indicates positive association (higher average values or greater exposure). − indicates negative association (lower average values or lower exposure). 0 indicates no association. ≠ indicate difference

^*^There was little variation in the relationship between PA patterns and self-esteem by gender

^a^Self-efficacy, Enjoyment, Perceived PA barriers, Perceived parent support for PA

^b^Parent support for PA, Sports/physically active lessons in past year, Screen devices in bedroom, Home PA equipment, Neighborhood safety

^c^School index; ≠ differences in the comparison between clusters, without the possibility of identifying the direction of the association

## Results

The searches resulted in 11,912 potentially relevant titles, of which 17 (11 from different data set) were identified and included in the review (Fig. 1). Table 1 summarizes each article included in the review. The year of publication varied from 2002 to 2017 and three studies were published in the last five years [26, 38, 40]. Four studies used data from two or more countries [13, 15, 34, 36] and a large number of studies were conducted in the United States [23, 24, 26, 37, 40]. All articles included were provide from high income countries. Exception for four studies [17, 36, 41, 42] all provided from macro-project data, and the exploratory data-based methods were applied cross-sectionally across all studies. Sample sizes ranged from 495 to 21,811 participants and most included a relatively equal distribution of boys and girls. Five studies identified cluster types in mixed-sex samples [23, 35, 37, 39, 42], and twelve studies according to sex [13, 15–17, 24–26, 34, 36, 38, 40, 41]. The age range was from six to 18 years old, with three studies involving children and adolescents [13, 17, 38], one only children [40], nine only adolescents [15, 16, 25, 26, 34, 35, 39, 41, 42], and four with an average age in the adolescent range [23, 24, 36, 37]. More instruments and behaviors outcomes information can be found elsewhere (see Table S7 supplementary material).

### Risk of bias assessment

The percentage of disagreement among the risk of bias evaluators was 34.7% (kappa = -0.25; 1.0), ranging from 5.9% to 64.7%. Only three studies [13, 23, 37] were considered to have a low risk of bias for all evaluated criteria and another study [34] showed moderate and low risk. The other studies showed a high risk of bias in at least one evaluated criterion (see Table S6 in supplementary material). Half of the included studies failed to achieve at least 60% of the eligible response (response rate), and a quarter of them had ≥ 80% of participants who completed the study. Almost all studies provided information that would allow researchers to replicate the PA and SB tool. According to Fig. 2, a high-risk selection bias was observed among studies.

### Behavior measurement and clusters variables

The classification of the instruments used to measure PA and SB is available in Table 2 and Supplementary material (Figure S1a and S1b). Objective measures were used in three studies [24, 34, 40] and one study [34], to evaluate PA and SB, respectively. Questionnaire was the most prevalent instrument used to measure PA (*n* = 11) [13, 16, 17, 25, 26, 35, 36, 38, 39, 41, 42], and SB (*n* = 13) [13, 16, 17, 24–26, 35, 36, 38–42]. All questionnaires applied [13, 16, 17, 24–26, 35, 36, 38–42] were consolidated or previously validated, and one [15] study used a diary, and two studies [23, 37] used recalls.

The most used variables for PA were Weekly PA (*n* = 11 articles [16, 24–26, 35, 36, 38–42]), followed by Weekly leisure-time PA (*n* = 6 articles) [13, 15–17, 25, 35] and Accelerometer Measured PA (*n* = 3 articles) [24, 34, 40]. PA in Physical Education classes and Daily PA were used by four [16, 23, 35, 37] and two [23, 37] articles, respectively. Five [13, 15, 17, 25, 39] articles used Leisure-time PA (i.e., yes or no) and one [26] used Muscle strengthening exercise (days/week) and Active sports team participation (number of modalities) as PA indicators (Table 3).

For SB, Weekly Computer Time was the most used variable (*n* = 10 studies) [15, 17, 23–25, 35–37, 41, 42] followed by Weekly Videogame Time (*n* = 9 studies) [17, 23–25, 35–37, 41, 42], Weekly TV Time (*n* = 9 studies) [15, 17, 23–25, 35, 37, 41, 42], and Weekly Non-screen Activities (*n* = 7 studies) [15, 17, 24, 36, 40–42]. Other studies used Weekly Phone Time (*n* = 4) [24, 36, 41, 42], Daily Stationary Time (*n* = 1) [34], Daily TV Time (*n* = 3) [13, 16, 26], Daily Computer Time (*n* = 3) [13, 16, 26] and Weekly Screen Time (*n* = 2) [24, 40]. Finally, indicators Weekly SB (screen and sit time [35]), Daily SB [38], Daily Videogame Time [16], and Daily Screen Time [39] were also used (Table 3).

### Description of the derived clusters

Studies included up to 16 input summary variables in cluster analysis. As presented in Table 1, cluster analysis (*n* = 11) [13, 15–17, 23, 34, 36–39, 41] was most commonly used approach to derive clusters, followed by latent class analysis (*n* = 3) [24, 26, 40], latent profile analysis (*n* = 2) [25, 42] and, principal component analysis (*n* = 1) [35]. A description of the cluster types defined by the reviewers and authors can be found in Table S8 supplementary material, and the prevalence and frequency of each cluster type identified in Table 4. The most prevalent clusters found in studies with the lowest risk of bias included “Low PA Low SB” and “High SB” for whole sample [23, 37], “Low SB” and “Low PA High/Low SB” for boys [13], and “Low PA Low SB”, “Low PA High SB” and “High PA Low SB” for girls [13].

Nine cluster types were identified for whole samples (i.e. boys and girls combined) (*n* = 5 studies) [23, 35, 37, 39, 42], these studies involved only adolescents and average adolescents’ age. The most frequently clusters identified in whole sample was “Low PA Low SB” (*n* = 4 studies) and “High PA High SB” (*n* = 3 studies). Otherwise, the most prevalent cluster types for whole samples were “Low PA Low SB” and “Low PA High/Low SB” and, highlighting that these was the clusters most prevalent in adolescents.

From studies that evaluated clusters according to sex (*n* = 12), twelve clusters were identified for boys and ten for girls. The most frequently cluster identified in boys was “High PA High SB” (*n* = 8 studies) and “Low PA Low SB” (*n* = 8 studies). Most prevalent cluster among boys were “High PA High SB”, “High PA Low SB”, and Low PA and Low SB. Girls’ most frequently clusters were “Low PA Low SB” (*n* = 8 studies), “Low PA High SB” (*n* = 6 studies), and ‘High PA Low SB” (*n* = 6 studies). Otherwise, the most prevalent clusters were “Low PA Low SB”, “Low PA High SB” and “High PA High SB”. Only one study was realized in children and procedure cluster analysis according to sex, the most prevalent cluster in both sexes were characterized by “Low PA Low SB”.

### Correlates and its association with clusters types

From the included studies a total of 31 correlates were investigated. The cluster correlates were sociodemographic factors [16, 17, 24, 36, 40]; adiposity indicators [13, 17, 24–26, 34, 36, 38, 40]; health risk behaviors [23]; and others factors, such as work and sleeping hours [17, 23, 37]; meeting PA guidelines [37]; and correlates of behavior at the individual [23, 40], interpersonal [40], and school level [40]. Table 5 presents all the correlates associated with cluster types.

The only study identified in children found null associations between school level, interpersonal and individual outcomes and cluster [40]. All information presented below, in subsequent paragraphs, refer to adolescents. Considering overweigh girls in the cluster “Low PA High/Low SB” presented negative [24] and positive [13] associations. Otherwise, at BMI outcome adolescents in cluster “High PA High SB” presented negative [34] and positive [38] associations.

Adolescents in “Low PA Low SB” clusters had higher odds of consuming alcohol [23], working [23] and lower odds of delinquency, wearing a seatbelt [23], sleeping ≥ 8 hours [23] and meeting PA guidelines in adolescence [37]. These results were found in studies with a low risk of bias. Boys in this cluster presented high odds to be overweight [24, 25] or obesity [26], low self-eficacy [40] and differences between age [16]. Girls in this cluster were older [16, 36], from North America [36], and are more likely to be obese [26].

Boys and girls in “High PA High SB” clusters, had higher BMI and were more likely to be obese in most of the tested associations [26, 38], whereas those in the “High PA Low SB” clusters had lower BMI and waist circumference and were less likely to be overweight or obese [24, 34].

Adolescents in "High PA" clusters had higher odds to work, sleeping ≥ 8 hours [23] and meeting PA guidelines in adolescence [37] and were less exposed to all health risk behaviors [23] and self-steem [23].

In the “Low SB” cluster, the results were similar, except for self-steem [23]. The associations found for "High PA" and “Low SB” were present in studies with low risk of bias.

In general, the correlates associated with clusters differed by sex. The similarities found, for the variables and the association direction, were: “High/Low PA Low SB cluster” vs age (differs); “High PA High SB cluster” vs obesity (positive); “High PA Low SB cluster” vs BMI and waist circumference (negative); “Low PA High SB cluster” vs obesity/overweight (positive); vs age and socioeconomic status/poverty (differs); “Low PA Low SB cluster” vs obesity (positive); vs age (differs); and “Low PA High/Low SB cluster” vs overweight (positive); vs age and socioeconomic status/poverty (differs).

## Discussion

This systematic review sought to provide comprehensive and up to date evidence on the clustering of SB and PA according to sex (identified using exploratory data-based methods) and their potential correlates. Nine, twelve and ten cluster types were identified for whole samples, boys, and girls, respectively. Boys were mostly allocated to the “High PA/High SB” clusters and girls to the “Low PA Low SB” clusters. Moreover, boys were more likely to accumulate time watching television time, using computer, and playing videogame and girls dedicate more time to paid work or housework [15, 17, 24, 36, 41]. Cluster types were associated with more than thirty different health-related correlates.

The risk of bias assessment identified methodological weaknesses in the studies, especially for the domains of sample selection and for withdrawal and dropouts. Few studies included samples representative of the target population, or were impacted by participant dropouts. Further, the number of participants who completed the study was often poorly reported across the studies. Having information on study response and dropout rates, as well as their reasons and the participant characteristics, allows a better interpretation of the results and the potential impact of selection bias. Future studies on clustering should therefore report the process of selection of participants, withdrawals and dropouts in a more comprehensive way.

Several cluster types with distinct combinations were identified for children and adolescents, and more than 70% of clusters included one negative behavior, corroborating with previous literature [6, 8, 12, 43]. In our review, girls were in clusters characterized by “Low PA High/Low SB” and “Low PA/Low SB”, while cluster types labelled “High PA Low SB”, followed by “High PA Low SB”, “High PA High SB” and “High PA” comprised more boys. Similar results from previous reviews showed that SB was inversely related to PA [44, 45] and high levels of PA coexisted with high and low levels of SB [6, 12, 43].

The predominance of unhealthy profiles in youths have been constantly reported in the literature [12, 14, 43] and, girls report lower levels of PA compared to boys [13, 14]. These differences can be explained by the way in which adolescents spend their time; boys spend more time being physically active PA and girls prefer to spend their time in socializing activities and in domestic tasks [46]. Moreover, motivational aspects such as the unwillingness [47] or discomfort from sweat and dirt [48] caused by PA contribute to girls being less physically active. Still, our results also demonstrated that girls were more often allocated to clusters characterized by large amounts of time in SB related to socializing components [15, 17, 24, 36, 41]. In contrast, boys were more likely to be in clusters characterized by large amounts of time using the computer and playing videogames [15–17, 24, 25, 36, 41], consistent with literature [15, 46, 49]. Studies have shown that different SB components have different effects on youths physical and mental health [50, 51]. For example, TV viewing was associated with worse physical health, quality of life and emotional problems, whereas interactive screen time (e.g. video game, social media and internet) showed negative psychological effects [50, 51]. These results suggest that policymakers, professionals, and parents should consider the type of youths’ screen time rather than only use-time. Also, is important to considered questionnaires to evaluated PA and SB once they are useful in collect data about variables context, whereas accelerometers provide more accurate info on time and intensity in each behavior.

In relation to the correlates of clusters, most studies included in this review evaluated adiposity indicators [13, 17, 24–26, 34, 36, 38, 40] followed by sociodemographic factors [16, 17, 24, 36, 40]. Few studies examined health risk behaviors [23]; sleeping hours [17, 23, 37], and individual [23, 40], interpersonal [40], and school level [40] correlates. Few associations were observed and most positive associations were found for at Health risk Behavior’s correlates provided from studies with low risk of bias. Briefly, clusters characterized by Low PA/Low SB presented lower probability to delinquency, wear seatbelt [23], sleeps ≥ 8 hours [23] and low self-eficacy [40], and cluster characterized by "High PA" presented less exposure for health risk behaviors [23] and self-steem [23]. However, further evidence is needed to clarify these relationships. Boys [24–26, 38] and girls [26, 38] in “Low PA Low SB” and “High PA High SB” clusters were more likely to have a higher BMI, or be overweight or obese. In contrast, better adiposity profiles were found when boys or girls were allocated to the “High PA Low SB” clusters [24, 34]. Physical inactivity and high time spent in SB are potential risks factors for increased adiposity [6, 12, 52] and their coexistence is linked to cumulative harmful effects to health [12, 53]. These findings emphasize the needed for the development of public policies strategies to promote PA and reduce SB simultaneously.

This was the first study to systematically review the clustering of PA and SB, and their associations with a comprehensive range of health correlates, in mixed-sex samples, and in boys and girls, separately. The search strategies were developed based on suggestion of experts on the theme which enabled the identification of many potential studies. This study also was able to identify and describe the behavior variables used to determine clusters. All these points advance the evidence base on clustering because previous reviews on cluster patterns were either not systematic [12], employed limited search strategies (i.e., limited combination of descriptors for PA and SB) [6, 12, 14] or limited the publications reviewed up to 2018 [6]. However, caution is needed when generalizing results: 1) the cluster type identified in this review were based on the authors’ interpretation based on descriptions reported by the studies’ authors. However, during the data extraction, a sequence of criteria and agreement between researchers was used to ensure that parsimonious information was obtained; 2) the wide range of PA and SB outcomes/variables made the synthesis of results challenging, however, the agreement process during the data extraction provided suitable information of the clusters types characterization; 3) we synthesized the direction of association and not the magnitude, which is important to understand for health-related variables.

The findings of this review have implications for future research examining the clustering of PA and SB. First, we emphasize that more studies examining clustering of PA and SB using data-driven exploratory methods should be conducted in children and adolescent populations from lower income countries, as none were found in this review and cluster types have been shown to differ according to socioeconomics variables [12, 54]. Second, more studies that employ and compare different exploratory data-based methods using the same data are needed to understand how different methods may yield different cluster patterns. Third, few studies provide sufficient detail regarding the analytic decisions taken to determine the optimal number of clusters and the reliability of the resulting cluster solution is rarely reported. Fourth, longitudinal studies are needed to identify how cluster patterns vary over time and to evaluate the effect of interventions on changing both PA and SB. Many large multi-component interventions have been implemented to change multiple behaviors simultaneously; however most studies are still using traditional methods approach of reporting changes in individual risk behaviors [55]. Fifth, studies that assess PA and SB using both device-based and self-report methods are needed to provide a richer understanding of behavior patterns and the contexts in which they occur. Further to this, analysis is needed to determine if cluster characterization (i.e., high/low PA, or high/low SB) varies according to whether behaviors are assessed using objective or questionnaire measurement tools. Finally, future cross-sectional and longitudinal studies examining the clustering of PA and SB should consider incorporating a wider range of modifiable correlates to better inform intervention strategies for behavior change.

We highlight that meta-analysis was not performed due to heterogeneity in measurements, analysis used and clusters types observed between studies. In order to conduct a meta-analysis, the cluster indicators and algorithms used in the clustering procedures would need to be standardized.

## Conclusion

In summary, the majority of cluster types had at least one unhealthy behavior in PA or SB indicators. Clusters differ in SB components in the profiles between boys and girls and high proportion of boys were allocated in cluster characterized by high PA. These demonstrate that different preventive approaches, tailored to boys and girls, need to be considered to improve children and adolescent lifestyles. Predominantly, clusters were associated with sociodemographic and adiposity correlates. Therefore, a better understanding of the modifiable correlates associated with PA and SB cluster types is needed to plan effective policies and interventions to improve youth lifestyles and subsequent health and wellbeing, and to develop guidelines considering simultaneously between behaviors once they together contribute to unhealthier health correlates.

## Supplementary Information

**Additional file 1:** **Table S1.** Prisma Checklist. **Table S2.** SWiM checklist. **Table S3.** Eligibility criteria. **Table S4.** Search of all strategy. **Table S5.** Adapted version of the Quality Assessment Tool for Quantitative Studies of Effective Public Health Practice Project (EPHPP). **Table S6.** Assessment of the bias risk of studies. **Table S7.** Clusters variables details before authors classifications. **Figure S1.** Instrument used and questionnaires classification according to each behavior. **Table S8.** Clusters detail.

## Data Availability

All data generated or analysed during this study are included in this published article.

## Abbreviations

PA: Physical activity
SB: Sedentary behavior
PRISMA: Preferred reporting items for systematic reviews and meta-analysis
SWiM: Synthesis without meta-analysis
EPHPP: Effective public health practice project

## References

[1] Matias TS, Lopes MVV, Mello GT, Silva KS (2019). Clustering of obesogenic behaviors and association with body image among Brazilian adolescents in the national school-based health survey (PeNSE 2015). Prev Med Rep.

[2] Dumuid D, Olds T, Lewis LK, Martin-Fernández JA, Barreira T, Broyles S (2018). The adiposity of children is associated with their lifestyle behaviours: a cluster analysis of school-aged children from 12 nations: Children’s adiposity relates to their lifestyle behaviours. Pediatr Obes.

[3] Dumuid D, Olds T, Lewis LK, Martin-Fernández JA, Katzmarzyk PT, Barreira T (2017). Health-Related Quality of Life and Lifestyle Behavior Clusters in School-Aged Children from 12 Countries. J Pediatr.

[4] Saunders TJ, Gray CE, Poitras VJ, Chaput JP, Janssen I, Katzmarzyk PT, et al. Combinations of physical activity, sedentary behaviour and sleep: relationships with health indicators in school-aged children and youth. Appl Physiol Nutr Metab. 2016;41(6 (Suppl. 3)):S283–93. 10.1139/apnm-2015-0626 27306434

[5] Carson V, Hunter S, Kuzik N, Gray CE, Poitras VJ, Chaput JP, et al. Systematic review of sedentary behaviour and health indicators in school-aged children and youth: an update. 2016;41:26. 10.1139/apnm-2015-0630 27306432

[6] Parker KE, Salmon J, Costigan SA, Villanueva K, Brown HL, Timperio A (2019). Activity-related behavior typologies in youth: a systematic review. Int J Behav Nutr Phys Act.

[7] Ferrar K, Chang C, Li M, Olds TS (2013). Adolescent Time Use Clusters: A Systematic Review. J Adolesc Health.

[8] Mello GT, Lopes MVV, Minatto G, Costa RMD, Matias TS, Guerra PH, Filho VCB, et al. Clustering of physical activity, diet and sedentary behavior among youth from low-, middle-, and high-income countries: a scoping review. Int J Env Res Public Health. 2021;18(20):10924. 10.3390/ijerph182010924 PMC8535526 34682670

[9] Chen ST, Liu Y, Hong JT, Tang Y, Cao ZB, Zhuang J (2018). Co-existence of physical activity and sedentary behavior among children and adolescents in Shanghai, China: do gender and age matter?. BMC Public Health.

[10] Guthold R, Cowan MJ, Autenrieth CS, Kann L, Riley LM (2010). Physical Activity and Sedentary Behavior Among Schoolchildren: A 34-Country Comparison. J Pediatr.

[11] Tremblay MS, Barnes JD, González SA, Katzmarzyk PT, Onywera VO, Reilly JJ, et al. Global Matrix 2.0: Report Card Grades on the Physical Activity of Children and Youth Comparing 38 Countries. J Phys Act Health. 2016;13(s2):S343–66. 10.1123/jpah.2016-0594 27848745

[12] Leech RM, McNaughton SA, Timperio A (2014). The clustering of diet, physical activity and sedentary behavior in children and adolescents: a review. Int J Behav Nutr Phys Act.

[13] te Velde SJ, De Bourdeaudhuij I, Thorsdottir I, Rasmussen M, Hagströmer M, Klepp KI (2007). Patterns in sedentary and exercise behaviors and associations with overweight in 9–14-year-old boys and girls - a cross-sectional study. BMC Public Health.

[14] D’Souza NJ, Kuswara K, Zheng M, Leech R, Downing KL, Lioret S, et al. A systematic review of lifestyle patterns and their association with adiposity in children aged 5–12 years. Obes Rev. 2020;21(8):e13029. 10.1111/obr.13029 32297464

[15] Gorely T, Marshall SJ, Biddle SJH, Cameron N (2007). Patterns of Sedentary Behaviour and Physical Activity Among Adolescents in the United Kingdom: Project STIL. J Behav Med.

[16] Spengler S, Mess F, Woll A (2015). Do Media Use and Physical Activity Compete in Adolescents? Results of the MoMo Study Zeeb H, organizador. PLOS ONE.

[17] Huang WY, Wong SH (2016). Time use clusters in children and their associations with sociodemographic factors. J Public Health.

[18] Mello GT, Silva KS, Matias TS, de Assis MAA, Borgatto AF (2021). Obesogenic Clusters Associated with Weight Status in Brazilian Adolescents of the Movimente School-Base Intervention. Int J Environ Res Public Health.

[19] Aubert S, Brazo-Sayavera J, González SA, Janssen I, Manyanga T, Oyeyemi AL (2021). Global prevalence of physical activity for children and adolescents; inconsistencies, research gaps, and recommendations: a narrative review. Int J Behav Nutr Phys Act.

[20] Rubín L, Gába A, Dygrýn J, Jakubec L, Materová E, Vencálek O (2020). Prevalence and correlates of adherence to the combined movement guidelines among Czech children and adolescents. BMC Public Health.

[21] Cooper AR, Goodman A, Page AS, Sherar LB, Esliger DW, van Sluijs EM (2015). Objectively measured physical activity and sedentary time in youth: the International children’s accelerometry database (ICAD). Int J Behav Nutr Phys Act.

[22] Jenkins GP, Evenson KR, Herring AH, Hales D, Stevens J (2017). Cardiometabolic Correlates of Physical Activity and Sedentary Patterns in U.S Youth. Med Sci Sports Exerc.

[23] Nelson MC (2006). Physical Activity and Sedentary Behavior Patterns Are Associated With Selected Adolescent Health Risk Behaviors. Pediatrics.

[24] Patnode CD, Lytle LA, Erickson DJ, Sirard JR, Barr-Anderson DJ, Story M (2011). Physical Activity and Sedentary Activity Patterns Among Children and Adolescents: A Latent Class Analysis Approach. J Phys Act Health.

[25] Melkevik O, Torsheim T, Rasmussen M. Patterns of screen-based sedentary behavior and physical activity and associations with overweight among Norwegian adolescents: a latent profile approach. Nor Epidemiol. 2011;20(1).

[26] Kim Y, Barreira TV, Kang M (2016). Concurrent Associations of Physical Activity and Screen-Based Sedentary Behavior on Obesity Among US Adolescents: A Latent Class Analysis. J Epidemiol.

[27] Morton KL, Atkin AJ, Corder K, Suhrcke M, Sluijs EMF (2016). The school environment and adolescent physical activity and sedentary behaviour: a mixed-studies systematic review. Obes Rev.

[28] Naylor PJ, Nettlefold L, Race D, Hoy C, Ashe MC, Wharf Higgins J (2015). Implementation of school based physical activity interventions: A systematic review. Prev Med.

[29] Russ LB, Webster CA, Beets MW, Phillips DS (2015). Systematic Review and Meta-Analysis of Multi-Component Interventions Through Schools to Increase Physical Activity. J Phys Act Health.

[30] Page MJ, McKenzie JE, Bossuyt PM, Boutron I, Hoffmann TC, Mulrow CD, The PRISMA (2020). statement: an updated guideline for reporting systematic reviews. BMJ.

[31] Page MJ, Moher D, Bossuyt PM, Boutron I, Hoffmann TC, Mulrow CD, PRISMA,  (2020). explanation and elaboration: updated guidance and exemplars for reporting systematic reviews. BMJ.

[32] Campbell M, McKenzie JE, Sowden A, Katikireddi SV, Brennan SE, Ellis S, et al. Synthesis without meta-analysis (SWiM) in systematic reviews: reporting guideline. BMJ. 2020;368:l6890. 10.1136/bmj.l6890 PMC7190266 31948937

[33] Thomas BH, Ciliska D, Dobbins M, Micucci S (2004). A Process for Systematically Reviewing the Literature: Providing the Research Evidence for Public Health Nursing Interventions. Worldviews Evid Based Nurs.

[34] De Bourdeaudhuij I, Verloigne M, Maes L, Van Lippevelde W, Chinapaw MJM, te Velde SJ (2013). Associations of physical activity and sedentary time with weight and weight status among 10- to 12-year-old boys and girls in Europe: a cluster analysis within the ENERGY project: Physical activity and sedentary time. Pediatr Obes.

[35] Lazarou C, Soteriades ES (2009). Physical Activity Patterns Among Preadolescent Children in Cyprus: The CYKIDS Study. J Phys Act Health.

[36] Marshall SJ, Biddle SJH, Sallis JF, McKenzie TL, Conway TL (2002). Clustering of Sedentary Behaviors and Physical Activity among Youth: A Cross-National Study. Pediatr Exerc Sci.

[37] Nelson MC, Gordon-Larsen P, Adair LS, Popkin BM (2005). Adolescent physical activity and sedentary behavior. Am J Prev Med.

[38] O’Neill A, Dowd K, O’Gorman C, Hannigan A, Walsh C, Purtill H (2017). Activity Profiles and the Associations With Weight Status in Population Studies of Young Children: Are There Gender Differences?. Pediatr Exerc Sci.

[39] Ramos P, Rivera F, Moreno C (2012). Análisis de clúster de la actividad física y las conductas sedentarias de los adolescentes españoles, correlación con la salud biopsicosocial. Rev Psicol Deporte.

[40] Taverno Ross SE, Dowda M, Dishman RK, Pate RR (2016). Classes of Physical Activity and Sedentary Behavior in 5th Grade Children. Am J Health Behav.

[41] Wang CKJ, Chia YHM, Quek JJ, Liu WC (2006). Patterns of physical activity, sedentary behaviors, and psychological determinants of physical activity among Singaporean school children. Int J Sport Exerc Psychol.

[42] Wang CKJ, Biddle SJH, Liu WC, Lim BSC (2012). A latent profile analysis of sedentary and physical activity patterns. J Public Health.

[43] Gubbels JS, van Assema P, Kremers SPJ (2013). Physical Activity, Sedentary Behavior, and Dietary Patterns among Children. Curr Nutr Rep.

[44] Platat C, Perrin AE, Oujaa M, Wagner A, Haan MC, Schlienger JL (2006). Diet and physical activity profiles in French preadolescents. Br J Nutr..

[45] Sallis JF, Prochaska JJ, Taylor WC (2000). A review of correlates of physical activity of children and adolescents. Med Sci Sports Exerc.

[46] Ferrar KE, Olds TS, Walters JL (2012). All the Stereotypes Confirmed: Differences in How Australian Boys and Girls Use Their Time. Health Educ Behav.

[47] Allender S, Cowburn G, Foster C (2006). Understanding participation in sport and physical activity among children and adults: a review of qualitative studies. Health Educ Res.

[48] Spencer RA, Rehman L, Kirk S (2015). Understanding gender norms, nutrition, and physical activity in adolescent girls: a scoping review. Int J Behav Nutr Phys Act.

[49] Marshall SJ, Gorely T, Biddle SJH (2006). A descriptive epidemiology of screen-based media use in youth: A review and critique. J Adolesc.

[50] Sanders T, Parker PD, del Pozo-Cruz B, Noetel M, Lonsdale C (2019). Type of screen time moderates effects on outcomes in 4013 children: evidence from the Longitudinal Study of Australian Children. Int J Behav Nutr Phys Act.

[51] Twenge JM, Farley E (2021). Not all screen time is created equal: associations with mental health vary by activity and gender. Soc Psychiatry Psychiatr Epidemiol.

[52] Carlson JA, Crespo NC, Sallis JF, Patterson RE, Elder JP (2012). Dietary-Related and Physical Activity-Related Predictors of Obesity in Children: A 2-Year Prospective Study. Child Obes.

[53] Van den Bulck J, Hofman A (2009). The television-to-exercise ratio is a predictor of overweight in adolescents: results from a prospective cohort study with a two year follow up. Prev Med.

[54] Matias TS, Silva KS, da Silva JA, de Mello GT, Salmon J (2018). Clustering of diet, physical activity and sedentary behavior among Brazilian adolescents in the national school - based health survey (PeNSE 2015). BMC Public Health.

[55] Prochaska JJ, Spring B, Nigg CR (2008). Multiple health behavior change research: An introduction and overview. Prev Med.

