# Supplementary material for: A systematic review of the clustering and correlates of physical activity and sedentary behavior among boys and girls
Source: BMC Public Health. 2023 Feb 21;23:372. doi: 10.1186/s12889-022-14869-0 (PMC9942368; doi:10.1186/s12889-022-14869-0)
Supplement: Supplementary file 1 — Additional file 1: Table S1. Prisma Checklist. Table S2. SWiM checklist. Table S3. Eligibility criteria. Table S4. Search of all strategy. Table S5. Adapted version of the Quality Assessment Tool for Quantitative Studies of Effective Public Health Practice Project (EPHPP). Table S6. Assessment of the bias risk of studies. Table S7. Clusters variables details before authors classifications. Figure S1. Instrument used and questionnaires classification according to each behavior. Table S8. Clusters detail. [file 12889_2022_14869_MOESM1_ESM.docx]

**A systematic review on the clustering and correlates of physical activity and sedentary behavior among boys and girls**

Gabrielli Thais de Mello^1*^, Cecília Bertuol^1^, Giseli Minatto^1^, Valter Cordeiro Barbosa Filho^5^, Brian Oldenburg^2,3,4^, Rebecca Maree Leech^6^, Kelly Samara Silva^1^

^1^ Research Center for Physical Activity and Health, Federal University of Santa Catarina, Florianópolis, SC, Brazil.

^2^ Melbourne School of Population and Global Health, University of Melbourne, Melbourne 3053, Australia;

^3^ Implementation Science Lab, Baker Heart and Diabetes Institute, Melbourne 3004, Australia

^4^ School of Psychology and Public Health, La Trobe University, Melbourne 3086, Australia

^5^ Federal Institute of Education, Science and Technology of Ceara, Aracati, Brazil.

^6^ Deakin University, Institute for Physical Activity and Nutrition (IPAN), Geelong, Australia.

**Table S1.** Prisma Checklist


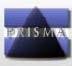


**PRISMA 2020 Checklist**

| **Section and Topic** | **Item #** | **Checklist item** | **Location where item**  **is reported** |
| --- | --- | --- | --- |
| **TITLE** | | |  |
| Title | 1 | Identify the report as a systematic review. | Page 1. Title |
| **ABSTRACT** | | |  |
| Abstract | 2 | See the PRISMA 2020 for Abstracts checklist. | Page 2 |
| **INTRODUCTION** | | |  |
| Rationale | 3 | Describe the rationale for the review in the context of existing knowledge. | Page 3 and 4 |
| Objectives | 4 | Provide an explicit statement of the objective(s) or question(s) the review addresses. | Page 4 and 5 |
| **METHODS** | | |  |
| Eligibility criteria | 5 | Specify the inclusion and exclusion criteria for the review and how studies were grouped for the syntheses. | Page 5. Topic eligibility criteria |
| Information sources | 6 | Specify all databases, registers, websites, organisations, reference lists and other sources searched or consulted to identify studies. Specify the date when each source was last searched or consulted. | Page 5 and 6. Topic Protocol and Search strategies and selection process |
| Search strategy | 7 | Present the full search strategies for all databases, registers and websites, including any filters and limits used. | Page 6. And, supplementary material Table S4 |
| Selection process | 8 | Specify the methods used to decide whether a study met the inclusion criteria of the review, including how many reviewers screened each record and each report retrieved, whether they worked independently, and if applicable, details of automation tools used in the process. | Page 6. Topic Search strategies and selection process |
| Data collection process | 9 | Specify the methods used to collect data from reports, including how many reviewers collected data from each report, whether they worked independently, any processes for obtaining or confirming data from study investigators, and if applicable, details of automation tools used in the process. | Page 6 and 7. Topic Data extraction and synthesis. |
| Data items | 10a | List and define all outcomes for which data were sought. Specify whether all results that were compatible with each outcome domain in each study were sought (e.g. for all measures, time points, analyses), and if not, the methods used to decide which results to collect. | Page 7. Topic Data extraction and synthesis. |
|  | 10b | List and define all other variables for which data were sought (e.g. participant and intervention characteristics, funding sources). Describe any assumptions made about any missing or unclear information. | Page 7. Topic Data extraction and synthesis. |
| Study risk of bias assessment | 11 | Specify the methods used to assess risk of bias in the included studies, including details of the tool(s) used, how many reviewers assessed each study and whether they worked independently, and if applicable, details of automation tools used in the process. | Page 6. Topic methodological quality assessment of include studies |
| Effect measures | 12 | Specify for each outcome the effect measure(s) (e.g. risk ratio, mean difference) used in the synthesis or presentation of results. | Not applicable |
| Synthesis methods | 13a | Describe the processes used to decide which studies were eligible for each synthesis (e.g. tabulating the study intervention characteristics and comparing against the planned groups for each synthesis (item #5)). | Page 7. Topic Data extraction and synthesis. |
|  | 13b | Describe any methods required to prepare the data for presentation or synthesis, such as handling of missing summary statistics, or data conversions. | Page 7. Topic Data extraction and synthesis. |
|  | 13c | Describe any methods used to tabulate or visually display results of individual studies and syntheses. | Page 7. Topic Data extraction and synthesis. |
|  | 13d | Describe any methods used to synthesize results and provide a rationale for the choice(s). If meta-analysis was performed, describe the model(s), method(s) to identify the presence and extent of statistical heterogeneity, and software package(s) used. | Page 7. Topic Data extraction and synthesis. |
|  | 13e | Describe any methods used to explore possible causes of heterogeneity among study results (e.g. subgroup analysis, meta-regression). | Page 7 and 8. Topic Data extraction and synthesis. |
|  | 13f | Describe any sensitivity analyses conducted to assess robustness of the synthesized results. | Page 7 and 8. Topic Data extraction and synthesis |
| Reporting bias assessment | 14 | Describe any methods used to assess risk of bias due to missing results in a synthesis (arising from reporting biases). | Page 6. Topic methodological quality assessment of include studies |
| Certainty assessment | 15 | Describe any methods used to assess certainty (or confidence) in the body of evidence for an outcome. | Page 7 and 8. Topic Data extraction and synthesis. |

| **Section and Topic** | **Item #** | **Checklist item** | **Location where item**  **is reported** |
| --- | --- | --- | --- |
| **RESULTS** | | |  |
| Study selection | 16a | Describe the results of the search and selection process, from the number of records identified in the search to the number of studies included in the review, ideally using a flow diagram. | Page 8. Topic results. |
|  | 16b | Cite studies that might appear to meet the inclusion criteria, but which were excluded, and explain why they were excluded. | Page 8. Topic results and Figure 1. |
| Study characteristics | 17 | Cite each included study and present its characteristics. | Page 8. Topic results. |
| Risk of bias in studies | 18 | Present assessments of risk of bias for each included study. | Page 9. Topic risk of bias assessment and Figure 2. |
| Results of individual studies | 19 | For all outcomes, present, for each study: (a) summary statistics for each group (where appropriate) and (b) an effect estimate and its precision (e.g. confidence/credible interval), ideally using structured tables or plots. | Page 8. |
| Results of syntheses | 20a | For each synthesis, briefly summarise the characteristics and risk of bias among contributing studies. | Page 9. Topic risk of bias assessment. |
|  | 20b | Present results of all statistical syntheses conducted. If meta-analysis was done, present for each the summary estimate and its precision (e.g. confidence/credible interval) and measures of statistical heterogeneity. If comparing groups, describe the direction of the effect. | Page 8 to 13. Topic results. |
|  | 20c | Present results of all investigations of possible causes of heterogeneity among study results. | Page 7 and 8. Topic data extraction and synthesis. |
|  | 20d | Present results of all sensitivity analyses conducted to assess the robustness of the synthesized results. | Page 7 and 8. Topic Data extraction and synthesis |
| Reporting biases | 21 | Present assessments of risk of bias due to missing results (arising from reporting biases) for each synthesis assessed. | Page 9. Risk of bias assessment. |
| Certainty of evidence | 22 | Present assessments of certainty (or confidence) in the body of evidence for each outcome assessed. | Page 7 and 8. Topic Data extraction and synthesis |
| **DISCUSSION** | | |  |
| Discussion | 23a | Provide a general interpretation of the results in the context of other evidence. | Page 13. |
|  | 23b | Discuss any limitations of the evidence included in the review. | Page 15 and 16. |
|  | 23c | Discuss any limitations of the review processes used. | Page 15 and 16. |
|  | 23d | Discuss implications of the results for practice, policy, and future research. | Page 16. |
| **OTHER INFORMATION** | | |  |
| Registration and protocol | 24a | Provide registration information for the review, including register name and registration number, or state that the review was not registered. | Page 5. Topic Protocol. |
|  | 24b | Indicate where the review protocol can be accessed, or state that a protocol was not prepared. | Page 5. Topic Protocol. |
|  | 24c | Describe and explain any amendments to information provided at registration or in the protocol. | Page 5. Topic Protocol. |
| Support | 25 | Describe sources of financial or non-financial support for the review, and the role of the funders or sponsors in the review. | Page 17. Topic Funding.. |
| Competing interests | 26 | Declare any competing interests of review authors. | Page 17. Topic competing interest. |
| Availability of data, code and other materials | 27 | Report which of the following are publicly available and where they can be found: template data collection forms; data extracted from included studies; data used for all analyses; analytic code; any other materials used in the review. | Supplementary material. |

*From:* Page MJ, McKenzie JE, Bossuyt PM, Boutron I, Hoffmann TC, Mulrow CD, et al. The PRISMA 2020 statement: an updated guideline for reporting systematic reviews. BMJ 2021;372:n71. doi: 10.1136/bmj.n71

For more information, visit: <http://www.prisma-statement.org/>

**Table S2.** SWiM checklist

The citation for the Synthesis Without Meta-analysis explanation and elaboration article is: Campbell M, McKenzie JE, Sowden A, Katikireddi SV, Brennan SE, Ellis S, Hartmann-Boyce J, Ryan R, Shepperd S, Thomas J, Welch V, Thomson H. Synthesis without meta-analysis (SWiM) in systematic reviews: reporting guideline BMJ 2020;368:l6890 <http://dx.doi.org/10.1136/bmj.l6890>

| **SWiM is intended to complement and be used as an extension to PRISMA** | | | |
| --- | --- | --- | --- |
| **SWiM reporting item** | **Item description** | **Page in manuscript where item is reported** | **Other*** |
| *Methods* | | | |
| **1** Grouping studies for synthesis | 1a) Provide a description of, and rationale for, the groups used in the synthesis (e.g., groupings of populations, interventions, outcomes, study design) | Page 5. Topic Protocol. |  |
|  | 1b) Detail and provide rationale for any changes made subsequent to the protocol in the groups used in the synthesis | Page 5. Topic Protocol. |  |
| **2** Describe the standardised metric and transformation methods used | Describe the standardised metric for each outcome. Explain why the metric(s) was chosen, and describe any methods used to transform the intervention effects, as reported in the study, to the standardised metric, citing any methodological guidance consulted | Page 6 and 7. Topic Data extraction and synthesis. |  |
| **3** Describe the synthesis methods | Describe and justify the methods used to synthesise the effects for each outcome when it was not possible to undertake a meta-analysis of effect estimates | Page 7 and 8. Topic Data extraction and synthesis. |  |
| **4** Criteria used to prioritise results for summary and synthesis | Where applicable, provide the criteria used, with supporting justification, to select the particular studies, or a particular study, for the main synthesis or to draw conclusions from the synthesis (e.g., based on study design, risk of bias assessments, directness in relation to the review question) | Page 7 and 8. Topic Data extraction and synthesis. |  |
| **SWiM reporting item** | **Item description** | **Page in manuscript where item is reported** | **Other*** |
| **5** Investigation of heterogeneity in reported effects | State the method(s) used to examine heterogeneity in reported effects when it was not possible to undertake a meta-analysis of effect estimates and its extensions to investigate heterogeneity | Page 7 and 8. Topic Data extraction and synthesis. |  |
| **6** Certainty of evidence | Describe the methods used to assess certainty of the synthesis findings | Page 7 and 8. Topic Data extraction and synthesis. |  |
| **7** Data presentation methods | Describe the graphical and tabular methods used to present the effects (e.g., tables, forest plots, harvest plots).  Specify key study characteristics (e.g., study design, risk of bias) used to order the studies, in the text and any tables or graphs, clearly referencing the studies included | Page 7 and 8. Topic Data extraction and synthesis. |  |
| *Results* | | | |
| **8** Reporting results | For each comparison and outcome, provide a description of the synthesised findings, and the certainty of the findings. Describe the result in language that is consistent with the question the synthesis addresses, and indicate which studies contribute to the synthesis | Page 8 to 13. |  |
| *Discussion* |  |  |  |
| **9** Limitations of the synthesis | Report the limitations of the synthesis methods used and/or the groupings used in the synthesis, and how these affect the conclusions that can be drawn in relation to the original review question | Page 16. |  |

PRISMA=Preferred Reporting Items for Systematic Reviews and Meta-Analyses.

*If the information is not provided in the systematic review, give details of where this information is available (e.g., protocol, other published papers (provide citation details), or website (provide the URL)).

**Table S3.** Eligibility criteria.

| **Items** | **Inclusion** | **Exclusion** |
| --- | --- | --- |
| Study | All studies design that applied exploratory data-based statistical procedures, considering cluster analysis (i.e., k-means), latent Class/Profile Analysis, and dimensionality reduction techniques (i.e., Principal Component Analysis and Factor Analysis); | Reviews, letters to editor, and conference abstracts |
| Outcome | analyzed simultaneously physical activity and sedentary behavior | other behaviors or variables (e.g., tobacco use, unhealthy eating, socioeconomic status) as part of the cluster patterns |
| Population | children and/or adolescents (aged 0–19 years, or reported means between these ages) | clinical populations (e.g., disabilities, metabolic and/or cardiovascular diseases, hospitalized or institutionalized populations) |
| Language | English, Portuguese, or Spanish | - |

**Table S4.** Search of all strategy

PUBMED

| **Search Group** | **Search Terms** |
| --- | --- |
| Physical Activity | sport* OR sports[mesh] OR sports OR "motor activity"[mesh] OR "motor activity" OR "physical activity" OR "physical activit*" OR exercise[mesh] OR exercise OR "exercise*" OR "physical exercise*" OR "exercise program*" OR "physical education" OR "physical fitness"[mesh] OR "physical fitness" OR "leisure time" OR "leisure activit*" OR "aerobic activity" OR "physical inactivity") |
| Sedentary Behavior | sedentarism OR sedentary OR "sedentary behavior" OR "sedentary behaviors" OR "sedentary behaviour" OR "sedentary behaviours" OR "sedentary lifestyle*" OR "sedentary lifestyle"[mesh] OR "sedentary lifestyle" OR television[mesh] OR television OR "television time" OR "television watch*" OR "TV watch*" OR "screen time" OR "screen viewing" OR "screen media" OR "media screen time" OR "time sitting" OR sitting OR "sitting time" OR computers[mesh] OR computers OR "computer time" OR "computer use" OR "video game*" |
| Diet Behavior | diet[mesh] OR diet OR "diet behavior" OR "diet behaviour" OR "diet consumption" OR "dietary intake" OR "unhealthy diet" OR "healthy diet"[mesh] OR "healthy diet" OR nutrition OR "food behavior" OR "feeding behavior"[mesh] OR "feeding behavior" OR "feeding behaviors" OR "feeding behaviour" OR "feeding behaviours" OR "eating behavior" OR "eating behaviors" OR "eating behaviour" OR "eating behaviours" OR "food consumption" OR "food choice" OR "food intake" OR "food habit" OR "food habits" OR "food preferences"[mesh] OR "food preferences" OR "unhealthy food" OR "nutritional quality" |
| Analysis | "cluster analysis"[mesh] OR "cluster analysis" OR cluster OR cluster* OR clustering OR co-occur OR co-occurrence OR "behavior pattern" OR "behavior patterns" OR "behaviour pattern" OR "behaviour patterns" OR "lifestyle pattern" OR "lifestyle patterns" OR "latent class" OR "factor analysis" OR "factorial analysis" OR simultaneity |
| Population | youth OR adolesce* OR adolescent[mesh] OR adolescent OR adolescent* OR adolescence OR student* OR students[mesh] OR students OR teen* OR teenage* OR schoolchildren OR child* OR child[mesh] OR child OR children[mesh] OR children |

Web of Science

| **Search Group** | **Search Terms** |
| --- | --- |
| Physical Activity | TS=(sport* OR sports OR "motor activity" OR "physical activity" OR "physical activit*" OR exercise OR "exercise*" OR "physical exercise*" OR "exercise program*" OR "physical education" OR "physical fitness" OR "leisure time" OR "leisure activit*" OR "aerobic activity" OR recreation OR "physical inactivity") |
| Sedentary Behavior | TS=(sedentarism OR sedentary OR "sedentary behavior" OR "sedentary behaviors" OR "sedentary behaviour" OR "sedentary behaviours" OR "sedentary lifestyle*" OR "sedentary lifestyle" OR television OR "television time" OR "television watch*" OR "TV watch*" OR "screen time" OR "screen viewing" OR "screen media" OR "media screen time" OR "time sitting" OR sitting OR "sitting time" OR computers OR "computer time" OR "computer use" OR "video game*") |
| Diet Behavior | TS=(diet OR "diet behavior" OR "diet behaviour" OR "diet consumption" OR "dietary intake" OR "unhealthy diet" OR "healthy diet" OR nutrition OR "food behavior" OR "feeding behavior" OR "feeding behaviors" OR "feeding behaviour" OR "feeding behaviours" OR "eating behavior" OR "eating behaviors" OR "eating behaviour" OR "eating behaviours" OR "food consumption" OR "food choice" OR "food intake" OR "food habit" OR "food habits" OR "food preferences" OR "unhealthy food" OR "nutritional quality") |
| Analysis | TS=("cluster analysis" OR cluster OR cluster* OR clustering OR co-occur OR co-occurrence OR "behavior pattern" OR "behavior patterns" OR "behaviour pattern" OR "behaviour patterns" OR "lifestyle pattern" OR "lifestyle patterns" OR "latent class" OR "factor analysis" OR "factorial analysis" OR simultaneity) |
| Population | TS=(youth OR adolesce* OR adolescent OR adolescent* OR adolescence OR student* OR students OR teen* OR teenage* OR schoolchildren OR child* OR child OR children) |

SCOPUS

| **Search Group** | **Search Terms** |
| --- | --- |
| Physical Activity | TITLE-ABS-KEY(sport* OR sports OR "motor activity" OR "physical activity" OR "physical activit*" OR exercise OR "exercise*" OR "physical exercise*" OR "exercise program*" OR "physical education" OR "physical fitness" OR "leisure time" OR "leisure activit*" OR "aerobic activity" OR recreation OR "physical inactivity") |
| Sedentary Behavior | TITLE-ABS-KEY(sedentarism OR sedentary OR "sedentary behavior" OR "sedentary behaviors" OR "sedentary behaviour" OR "sedentary behaviours" OR "sedentary lifestyle*" OR "sedentary lifestyle" OR television OR "television time" OR "television watch*" OR "TV watch*" OR "screen time" OR "screen viewing" OR "screen media" OR "media screen time" OR "time sitting" OR sitting OR "sitting time" OR computers OR "computer time" OR "computer use" OR "video game*") |
| Diet Behavior | TITLE-ABS-KEY(diet OR "diet behavior" OR "diet behaviour" OR "diet consumption" OR "dietary intake" OR "unhealthy diet" OR "healthy diet" OR nutrition OR "food behavior" OR "feeding behavior" OR "feeding behaviors" OR "feeding behaviour" OR "feeding behaviours" OR "eating behavior" OR "eating behaviors" OR "eating behaviour" OR "eating behaviours" OR "food consumption" OR "food choice" OR "food intake" OR "food habit" OR "food habits" OR "food preferences" OR "unhealthy food" OR "nutritional quality") |
| Analysis | TITLE-ABS-KEY("cluster analysis" OR cluster OR cluster* OR clustering OR co-occur OR co-occurrence OR "behavior pattern" OR "behavior patterns" OR "behaviour pattern" OR "behaviour patterns" OR "lifestyle pattern" OR "lifestyle patterns" OR "latent class" OR "factor analysis" OR "factorial analysis" OR simultaneity) |
| Population | TITLE-ABS-KEY(youth OR adolesce* OR adolescent OR adolescent* OR adolescence OR student* OR students OR teen* OR teenage* OR schoolchildren OR child* OR child OR children) |

LILACS, MEDLINE AND PSYCINFO

| **Search Group** | **Search Terms** |
| --- | --- |
| Physical Activity | (sport OR sports OR "motor activity" OR "physical activity" OR "physical activities" OR exercise OR exercises OR "physical exercise" OR "exercise program*" OR "physical education" OR "physical fitness" OR "leisure time" OR "leisure activity" OR "leisure activities" OR "aerobic activity" OR recreation OR "physical inactivity") |
| Sedentary Behavior | (sedentarism OR sedentary OR "sedentary behavior" OR "sedentary behaviors" OR "sedentary behaviour" OR "sedentary behaviours" OR "sedentary lifestyles" OR "sedentary lifestyle" OR television OR "television time" OR "television watch" OR "television watches" OR "TV watch" OR "TV watching" OR "TC watches" OR "screen time" OR "screen viewing" OR "screen media" OR "media screen time" OR "time sitting" OR sitting OR "sitting time" OR computers OR "computer time" OR "computer use" OR "video game" OR "video games") |
| Diet Behavior | (diet OR "diet behavior" OR "diet behaviour" OR "diet consumption" OR "dietary intake" OR "unhealthy diet" OR "healthy diet" OR nutrition OR "food behavior" OR "feeding behavior" OR "feeding behaviors" OR "feeding behaviour" OR "feeding behaviours" OR "eating behavior" OR "eating behaviors" OR "eating behaviour" OR "eating behaviours" OR "food consumption" OR "food choice" OR "food intake" OR "food habit" OR "food habits" OR "food preferences" OR "unhealthy food" OR "nutritional quality") |
| Clustering | ("cluster analysis" OR cluster OR cluster* OR clustering OR co-occur OR co-occurrence OR "behavior pattern" OR "behavior patterns" OR "behaviour pattern" OR "behaviour patterns" OR "lifestyle pattern" OR "lifestyle patterns" OR "latent class" OR "factor analysis" OR "factorial analysis" OR simultaneity) |
| Population | (youth OR adolesce* OR adolescent OR adolescent* OR adolescence OR student* OR students OR teen* OR teenage* OR schoolchildren OR child* OR child OR children) |

**Table S5.** Adapted version of the Quality Assessment Tool for Quantitative Studies of Effective Public Health Practice Project (EPHPP).

| **Domain** | **Question** | **Classification** | |
| --- | --- | --- | --- |
| 1) Selection bias | Are the individuals selected to participate in the study likely to be representative of the target population? | ≥80% = *strong* or 1  79 - 60% = *moderate* or 0  ≤60% = *weak* or -1 | |
| 2) Study design | Is there a description of the representativeness of the sample? | Yes = 1  No = 0 | *Strong:* 1 in all three items.  *Moderate:* for combinations: 1-1-0, 1-0-1, 1-0-0, and 0-0-1.  *Weak:* for all other combinations. |
|  | Was the sampling method described? | Yes = 1  No = 0 |  |
|  | Was the method appropriate? | Random = 1  Not described = 0  Convenience = -1 |  |
| 3) information about instruments to evaluate PA and SB and information that would enable reproducing PA and SB assessment | Is there a prior validation report of the tool? | Yes = 1  No = 0 | *Studies using accelerometer to measure PA and/or SB were assigned score "1", that is, it was considered that there was a previous validation report of the instrument.  *Strong:* for 1 in both outcome items.  *Weak:* for all other combinations. |
|  | Is there information that makes it possible to replicate the tool? | Yes = 1  No = 0 |  |
| 4) Flow of people throughout the study and percentage of participants completing the study | Were withdrawals and drop-outs reported in terms of numbers and/or reasons per group? | Yes = 1  No = 0 | *Strong:* was applied for 1 in both items or 0 and 1. M*oderate:* for combinations 1 and 0 or 0 and 0.  *Weak:* for all other combinations. |
|  | Indicate the percentage of participants completing the study? | ≥80% = 1 or *strong;*  60–79% = 0 or *moderate*; ≤59%= -1 or *weak*. |  |

**Table S6.** Assessment of the bias risk of studies.

| **Study** | **Selection bias** | | **Study design** | | | | **Assessment tool** | | | | | | **Withdrawals and drop-outs** | | |
| --- | --- | --- | --- | --- | --- | --- | --- | --- | --- | --- | --- | --- | --- | --- | --- |
|  |  |  |  |  |  |  | **PA** | | | **SB** | | |  |  |  |
|  | **Q1** | **Bias** | **Q2** | **Q3** | **Q4** | **Bias** | **Q5** | **Q6** | **Bias** | **Q5** | **Q6** | **Bias** | **Q7** | **Q8** | **Bias** |
| De Bourdeaudhuij (2013) | 0 | Moderate | 1 | 1 | 1 | Strong | 1 | 1 | Strong | 1 | 1 | Strong | 1 | 0 | Moderate |
| Gorely (2007) | -1 | Weak | 1 | 1 | 1 | Strong | 1 | 1 | Strong | 1 | 1 | Strong | 1 | -1 | Weak |
| Huang (2015) | -1 | Weak | 1 | 1 | 1 | Strong | 0 | 1 | Weak | 1 | 1 | Strong | 1 | 1 | Strong |
| Kim (2016) | 1 | Strong | 1 | 1 | 1 | Strong | 0 | 1 | Weak | 0 | 1 | Weak | 1 | 1 | Strong |
| Lazarou (2009) | ? | Weak | 0 | -1 | 0 | Weak | 1 | 1 | Strong | 1 | 1 | Strong | 1 | -1 | Weak |
| Marshall (2002) | 1 | Strong | 1 | -1 | 1 | Weak | 1 | 1 | Strong | 0 | 1 | Weak | 1 | 1 | Strong |
| Melkevik (2010) | -1 | Weak | 1 | 1 | 1 | Strong | 1 | 1 | Strong | 1 | 1 | Strong | 1 | 0 | Moderate |
| Nelson (2005) | 1 | Strong | 1 | 1 | 1 | Strong | 1 | 1 | Strong | 1 | 1 | Strong | 1 | 1 | Strong |
| Nelson (2006) | 1 | Strong | 1 | 1 | 1 | Strong | 1 | 1 | Strong | 1 | 1 | Strong | 1 | 1 | Strong |
| O'Neill (2016) | 1 | Strong | 1 | 1 | 1 | Strong | 0 | 1 | Weak | 0 | 1 | Weak | 1 | -1 | Weak |
| Patnode (2011) | 1/?* | Strong/Weak | 1 | 1 | -1 | Weak | 1 | 1 | Strong | 1 | 1 | Strong | 0 | 0 | Moderate |
| Ramos (2012) | ? | Weak | 1 | 1 | 1 | Strong | 1 | 1 | Strong | 1 | 1 | Strong | 0 | 0 | Moderate |
| Spengler (2015) | -1 | Weak | 1 | 1 | 1 | Strong | 1 | 1 | Strong | 0 | 1 | Weak | 1 | -1 | Weak |
| Taverno (2016) | -1 | Weak | 1 | 1 | 0 | Moderate | 1 | 1 | Strong | 1 | 1 | Strong | 1 | -1 | Weak |
| Te Velde (2007) | 1 | Strong | 1 | 1 | 1 | Strong | 1 | 1 | Strong | 1 | 1 | Strong | 1 | 1 | Strong |
| Wang (2006) | ? | Weak | 0 | 0 | 0 | Weak | 1 | 1 | Strong | 1 | 1 | Strong | 0 | 0 | Moderate |
| Wang (2012) | ? | Weak | -1 | 0 | 1 | Weak | 1 | 1 | Strong | 1 | 1 | Strong | 0 | 0 | Moderate |

PA: physical activity; SB: sedentary behavior; ?: impossible to determine; * IDEA study = 1 and ECHO study = ?;

Q1: Are the individuals selected to participate in the study likely to be representative of the target population?; Q2: Is there a description of the representativeness of the sample?; Q3: Was the sampling method described?; Q4: Was the method appropriate?; Q5: Is there a prior validation report of the tool?; Q6: Is there information that makes it possible to replicate the tool?; Q7: Were withdrawals and drop-outs reported in terms of numbers and/or reasons per group?; Q8: Indicate the percentage of participants completing the study.

**Table S7.** Clusters variables details before authors classifications.

| **First author (publication year)** | **Clusters outcomes (indicator; instrument; variable treatment / unit of measurement)** | |
| --- | --- | --- |
|  | **PA** | **SB** |
| De Bourdeaudhuij (2013) | Accelerometer *Indicator:* minutes of MVPA | Accelerometer *Indicator:* minutes of sedentary time |
| Gorely (2007) | Self-report diary of ‘‘free-time’’ *Indicator:* sports or exercises during leisure-time (weekly) | Self-report diary of "free-time" *Indicators:* television/video viewing, computer use, socialising behaviours, homework, and working (weekly) |
| Huang (2015) | Questionnaire (CLASS-C) *Indicator:* min/day spent on MVPA in leisure-time (weekly) | Questionnaire (CLASS-C) *Indicators:* time spent on doing homework, watching TV, playing electronic games, using the Internet, reading, listening to music and engaging in socializing behavior (weekly) |
| Kim (2016) | Self-report Indicators: frequency of MVPA, sports team participation, and muscle-strengthening exercise (days/week) | Self-report Indicators: hours/day watching TV, and using a computer |
| Lazarou (2009) | Semi-quantitative questionnaire *Indicators:* frequency and duration of everyday physical activities (physical activity and sports after school + home chores and outside home chores, aerobics, gymnastics, sports + sports for all, afterschool activities [except sports]) | Semi-quantitative questionnaire *Indicators:* frequency and duration of everyday sedentary activities (video, electronic games, and computers + watching TV, video, and DVD + homework and private lessons + theater cinema, use of mobile phone + afternoon sleep, fewer private lessons) |
| Marshall (2002) | SAPAC: Modified version. *Indicator:* metabolic equivalent values, classified as: no, low, moderate, and high activity (weekly) | SAPAC Modified version.  *Indicators:* time spent on the computer/internet, playing video games, doing homework, reading (not for school), sitting and talking/listening to music, and talking on the telephone (weekly) |
| Melkevik (2010) | Self-report *Indicators:* leisure time: VPA and MVPA | Self-report *Indicators:* time spent watching television (including videos), playing PC-games or TV-games, and using a computer (weekly) |
| Nelson (2005) | 7-day recall questionnaire *Indicators:* Week Bouts (Hobbies, housework, skating, sports, exercise). Number for year (school academic clubs, school team sports, school individual sports); Weekdays (school physical education). Llikelihood of playing sport with a parent. Likelihood of using a recreation center. | 7-day recall questionnaire *Indicators:* Week bouts (Hang out). Week hours (television viewing, video viewing, video game playing. Likelihood of making own television decisions |
| Nelson (2006) | 7-day recall questionnaire *Indicators:* Week bouts (Hobbies, housework, skating, sports, exercise). Number for year (school academic clubs, school team sports, school individual sports). Weekdays (school physical education). Llikelihood of playing sport with a parent. Likelihood of using a recreation center. | 7-day recall questionnaire *Indicators:* Week bouts (Hang out). Week hours (television viewing, video viewing, video game playing. Likelihood of making own television decisions |
| O'Neill (2016) | Self and parental report *Indicators:* active favourite hobby, and travel to school (active commuting) | Self-report *Indicators:* total daily time in SB (watching TV, using the computer, time spent playing video games, and reading time) |
| Patnode (2011) | Accelerometer, and 3-Day Physical Activity Recall *Indicators:* MVPA on weekdays and weekend days, traditional sports, fitness activities, other sports and physical activities, and chores/work | Self-administered Project EAT Items adapted  *Indicators*: time spent watching television, watching DVDs or videos, reading/homework, Nintendo/Play Station/computer games, internet/computers, and talking on the phone or cell phone/text messaging for both typical weekdays and weekend days |
| Ramos (2012) | HBSC questionnaire *Indicators:* MVPA recommendation, and VPA (weekly) | HBSC questionnaire *Indicators*: daily screen time (watching television, playing with the computer or the console, and using the computer) |
| Spengler (2015) | Questionnaire MoMo-PAQ *Indicators:* weekly duration of elective PA at school, PA at sports clubs, and leisure time PA outside of sports clubs | KiGGS telephone interview. *Indicators:* daily time spent watching television or video, using a computer, and playing console games |
| Taverno Ross (2016) | Accelerometer, and PAC instrument *Indicators:* MVPA, individual physical activities, team sports, lifestyle activities, wheel activities (weekly) | PAC instrument *Indicators:* educational sedentary, and electronic media (weekly) |
| te Velde (2007) | Pro Children Project website questionnaire. *Indicator:* hours spent on leisure-time PA (weekly) | Pro Children Project website questionnaire. *Indicators:* hours/day spent on usual TV viewing, PC use, and TV viewing during dinner |
| Wang (2006) | Modified SAPAC *Indicators:* minutes/week and metabolic equivalent (MET) values of 32 physical activities (physical activity levels categorized as inactive, low, moderate, and high) | Modified SAPAC *Indicators:* time spent on computer/internet, video game, time studying, reading, sitting/talking, using telephone, and watching TV (weekly) |
| Wang (2012) | SAPAC, and a 7-day PA recall questionnaire *Indicators:* minutes/week of 28 physical activities | SAPAC, and a 7-day PA recall questionnaire *Indicators:* time spent on computer/internet, video game, time studying, reading, sitting/talking, using telephone, and watching TV (weekly) |

(A) Instrument Types

(B) Questionnaires Classification

**Figure S1.** Instrument used and questionnaires classification according to each behavior.

| **Table S8. Clusters detail** | | |
| --- | --- | --- |
| **Author (publication year)** | **Clusters types identified in paper (N / %)** | **Cluster types defined by review authors** |
| De Bourdeaudhuij (2013) | Boys (n= 361) Cluster 1 (n= 100, 27.70%) Cluster 2 (n= 72, 19.95%) Cluster 3 (n= 107, 29.64%) Cluster 4 (n= 82, 22.71%) Girls (n= 405) Cluster 1 (n= 97, 23.95%) Cluster 2 (n= 85, 20,99%) Cluster 3 (n= 119, 29.38%) Cluster 4 (n= 104, 25.68%) | Girls  Cluster 1 - Low PA High SB Cluster 2 High Pa Low SB Cluster 3 - Low PA Low SB Cluster 4 - High SB  Boys  Cluster 1 - Low PA High SB Cluster 2 - High Pa Low SB Cluster 3 - Low PA Low SB Cluster 4 - High PA High SB |
| Gorely (2007) | Boys (n= 484) Cluster 1 – sedentary homeworkers (n= 93, 19.2%) Cluster 2 – semi-active socializers (n= 97, 20.0%) Cluster 3 – sedentary television watchers (n= 144, 30%) Cluster 4 – actives (n= 75, 15.4%) Cluster 5 – sedentary computer users (n= 75, 15.4%)  Girls (n= 785) Cluster 1 – sedentary homeworkers (n= 198, 25.2%) Cluster 2 – sedentary socializers (n= 206, 26.2%) Cluster 3 – sedentary television watchers (n= 181, 23.1%) Cluster 4 – actives (n= 114, 14.5%) Cluster 5 – sedentary workers (n= 86, 11.0%) | Girls  Cluster 1 – Low PA High/Low SB  Cluster 2 – Low PA High/Low SB  Cluster 3 – Low PA High/Low SB  Cluster 4 – High PA Low SB Cluster 5 – Low PA High/Low SB Boys Cluster 1 – Low PA High/Low SB Cluster 2 – Low PA High/Low SB  Cluster 3 – Low PA High/Low SB  Cluster 4 – High PA Low SB Cluster 5 – Low PA High/Low SB |
| Huang (2015) | Boys (n= 471) Cluster 1 – actives (n= 43, 9.1%) Cluster 2 – inactive (n= 280, 59.4%) Cluster 3 – sedentary homeworkers (n= 22, 4.7%) Cluster 4 – sedentary TV viewers (n= 78, 16.6%) Cluster 5 – Sedentary games players (n= 48, 10.2%) Girls (n= 480) Cluster 1 – actives (n= 57, 11.9%) Cluster 2 – uninvolved inactive (n= 190, 39.5%) Cluster 3 – sedentary homeworkers (n= 54, 11.3%) Cluster 4 – sedentary TV viewers (n= 41, 8.5%) Cluster 5 – sedentary socializers (n= 138, 28.8%) | Girls  Cluster 1 – s High PA Low SB  Cluster 2 – Low PA Low SB Cluster 3 – High PA High SB Cluster 4 – High SB Cluster 5 – Low PA High SB Boys  Cluster 1 – High PA Low SB  Cluster 2 – Low PA Low SB Cluster 3 – High/Low SB Cluster 4 – High PA High SB Cluster 5 – High SB |
| Kim (2016) | Boys (n= 6,109) Class 1 – high PA and high SB (n= 1240, 20.3%) Class 2 – high PA and low SB (n= 2351, 38.5%) Class 3 – low PA and high SB (n= 471, 7.7%) Class 4 – low PA and low SB (n= 2047, 33.5%) Girls (n= 5,972) Class 1 – high PA and high SB (n= 1051, 17.6%) Class 2 – high PA and low SB (n= 1380, 23.1%) Class 3 – low PA and high SB (n= 1577, 26.4%) Class 4 – low PA and low SB (n= 1964, 32.9%) | Equal to boys and girls Class 1 - High PA/ High SB  Class 2 - High PA / Low SB  Class 3 - Low PA / High SB Class 4 - Low PA / Low SB |
| Lazarou (2009) | 3 PA factors / 5 SB factors Factor 1 – physical activity, and sports after school (significantly higher for boys compared to girls) Factor 2 – video, electronic games, and computers (significantly higher for boys compared to girls)  Factor 3 – watching TV, video, and DVD  Factor 4 – homework, and private lessons (significantly higher for girls compared to boys) Factor 5 – home chores, and outside home chores, aerobics, gymnastics, sports  Factor 6 – theater cinema, use of mobile phone (significantly higher for girls compared to boys)  Factor 7 – afternoon sleep, less private lessons  Factor 8 – sports for all, after-school activities (except sports) | 3 PA factors - High PA  5 SB factors - High SB |
| Marshall (2002) | Boys (n= 819) Cluster 1 – techno-actives (n= 333, 40.7%) Cluster 2 – non-socializing actives (n= 383, 46.7%) Cluster 3 – uninvolved inactives (n= 103, 12.6%) Girls (n= 1,570)  Cluster 1 – sociable actives (n= 243, 15.5%) Cluster 2 – non-socializing actives (n= 562, 35.8%) Cluster 3 – uninvolved inactives (n= 765, 48.7%) | Boys Cluster 1 - High PA High SB Cluster 2 - High PA Low SB Cluster 3 - Low PA Low SB  Girls Cluster 1 - High PA High SB  Cluster 2 - High PA Low SB Cluster 3 - Low PA Low SB |
| Melkevik (2010) | Boys (n= 2,520) Cluster 1 – moderate SBSB and very high PA (n= 605, 24%) Cluster 2 – moderate SBSB and high PA (n= 630, 25%) Cluster 3 – moderate SBSB and moderate PA (n= 580, 23%) Cluster 4 – low SBSB and low PA (n= 302, 12%) Cluster 5 – high SBSB and low PA (n= 353, 14%) Cluster 6 – very high SBSB and low PA (n= 50, 2%) Girls (n= 2,328) Cluster 1 – moderate SBSB and very high PA (n= 303, 13%) Cluster 2 – moderate SBSB and high PA (n= 466, 20%) Cluster 3 – moderate SBSB and moderate PA (n= 628, 27%) Cluster 4 – moderate SBSB and low PA (n= 256, 11%) Cluster 5 – moderate SBSB (no gaming) and low PA (n= 419, 18%) Cluster 6 – high SBSB and moderate PA (n= 256,11%) | Boys Cluster 1 - High PA - High PA Cluster 2 - High PA Cluster 3 - none Cluster 4 - Low PA Low SB Cluster 5 - Low PA High SB Cluster 6- Low PA High SB Girls Cluster 1 - High PA Cluster 2 - High PA Cluster 3 - none Cluster 4 - Low PA Cluster 5 - Low PA Cluster 6 - High SB |
| Nelson (2005) | Cluster 1 – TV/video and gaming (n= 2494, 20.9%) Cluster 2 – skaters and gamers (n= 1119, 9.4%) Cluster 3 – sports with parents (n= 1681, 14.1%) Cluster 4 – uses recreation center (n= 1309, 10.9%) Cluster 5 – limited TV decisions (n= 1522, 12.7%) Cluster 6 – reports few activities (n= 2897, 24.2%) Cluster 7 – active in school (n= 935, 7.8%) | Cluster 1 - High SB Cluster 2 - High PA High SB Cluster 3 - High PA Cluster 4- High PA Cluster 5 - Low SB Cluster 6- Low PA Low SB Cluster 7- High PA |
| Nelson (2006) | Cluster 1 – TV/video and gaming (n= 2494, 20.9%) Cluster 2 – skaters and gamers (n= 1119, 9.4%) Cluster 3 – sports with parents (n= 1681, 14.1%) Cluster 4 – uses recreation center (n= 1309, 10.9%) Cluster 5 – limited TV decisions (n= 1522, 12.7%) Cluster 6 – reports few activities (n= 2897, 24.2%) Cluster 7 – active in school (n= 935, 7.8%) | Cluster 1 - High SB Cluster 2 - High PA High SB Cluster 3 - High PA Cluster 4- High PA Cluster 5 - Low SB Cluster 6- Low PA Low SB Cluster 7- High PA |
| O'Neill (2016) | Boys (n= 4,298)* Cluster 1 – high PA and 4.03 mean hours of SB (n= 1924, 43.9%) Cluster 2 – high PA and 4.24 mean hours of SB (n= 807, 18.4%) Cluster 3 – low PA and 4.57 mean hours of SB (n= 578, 13.2%)  Cluster 4 – low PA and 4.39 mean hours of SB (n= 989, 22.6%)  * 83 cases were excluded due to missing data on one or more of the above variables   Girls (did not found coherent profiles) | Boys Cluster 1 - High PA High SB  Cluster 2 - High PA High SB  Cluster 3 - Low PA High SB  Cluster 4 - Low PA High SB |
| Patnode (2011) | Boys (n= 352) Cluster 1 – active (n= 148, 42.1%) Cluster 2 – sedentary (n= 88, 24.9%) Cluster 3 – low media/moderate activity (n= 116, 33.0%) Girls (n= 368) Cluster 1 – active (n= 69, 18.7%) Cluster 2 – sedentary (n= 175, 47.6%) Cluster 3 – low media/functional activity (n= 124, 33.7%) | Boys  Cluster 1 - High PA Low SB Cluster 2 - Low PA High/Low SB - Cluster 3 - Low PA Low SB Girls  Cluster 1 - High PA and High/Low SB  Cluster 2 - Low PA high SB  Cluster 3 - Low PA High/Low SB |
| Ramos (2012) | Cluster 1 – high MVPA/VPA and low SB (n= 5042, 25.4%) Cluster 2 – high SB and low MVPA/VPA (n= 4404, 22.1%) Cluster 3 – low MVPA/VPA and low SB (n= 10889, 52.5%) | Cluster 1 - High PA Low Cluster 2 - Low PA High SB  Cluster 3 - Low PA Low SB |
| Spengler (2015) | Boys (n= 1,031) Cluster 1 (n= 343, 33.3%)  Cluster 2 (n= 126, 12.2%) Cluster 3 (n= 147, 14.3%) Cluster 4 (n= 50, 4.8%) Cluster 5 (n= 53, 5.2%) Cluster 6 (n= 65, 6.3%) Cluster 7 (n= 50, 4.8%) Cluster 8 (n= 197, 19.1%)  Girls (n= 1,052)  Cluster 1 (n= 443,42.1%) Cluster 2 (n= 97, 9.2%) Cluster 3 (n= 164, 15.6%) Cluster 4 (n= 65, 6.2%) Cluster 5 (n= 54, 5.1%) Cluster 6 (n= 105, 10.0%) Cluster 7 (n= 124, 11.8%) | Boys (1) Low PA Low SB  (2) Low PA High/Low SB  (3) Low PA High/Low SB  (4) Low PA High SB  (5) High/Low PA  (6) High/Low PA  (7) High/Low PA  (8) High/Low PA Low SB  Girls (1) Low PA Low SB  (2) Low PA High/Low SB (3) Low PA High/Low SB  (4) Low PA High SB (5) High/Low PA Low SB  (6) High/Low PA Low SB  (7) Low PA Low SB |
| Taverno (2016) | Boys (n= 221) Cluster 1 – low PA and low SB (n= 156, 70.6%) Cluster 2 – moderate PA and high SB (n= 34, 15.4%) Cluster 3 – high PA and high SB (n= 31, 14.0%) Girls (n= 274)  Cluster 1 – low PA and low SB (n= 149, 54.4%) Cluster 2 – moderate PA and high SB (n= 90, 32,8%) Cluster 3 – high PA (n= 35, 12.8%) | Boys Class 1 - Low PA Low SB  Class 2 - High SB  Class 3 - High PA High SB  Girls  Class 1 - Low PA Low SB  Class 2 - High SB  Class 3 - High PA |
| Te velde (2007) | Boys (n= 6,255) Cluster 1 – healthy behavior pattern (n= 2624, 42.0%) Cluster 2 – high TV viewers (n= 1100, 17.6%) Cluster 3 – mixed pattern (n= 1494, 23.9%) Cluster 4 – high PC users (n= 601, 9.6%) Cluster 5 – unhealthy behavior pattern (n= 436, 6.9%) Girls (n= 6,283)  Cluster 1 – healthy behavior pattern (n= 1337, 21.3%)  Cluster 2 – high TV viewers (n= 1339, 21.3%) Cluster 3 – low SB and low physical exercise behavior (n= 2794, 44.5%) Cluster 4 – high PC users (n= 584, 9.3%) Cluster 5 – high SB and high physical exercise (n= 229, 3.6%) | Boys  Cluster 1 - Low SB  Cluster 2 - High PA High SB  Cluster 3- Low PA High/Low SB  Cluster 4 - Low PA High/Low SB  Cluster 5 - Low PA High SB  Girls  Cluster 1 - High PA Low SB  Cluster 2 - Low PA High SB Cluster 3 - Low PA Low SB  Cluster 4 - Low PA High/Low Cluster 5 - High PA High SB |
| Wang (2006) | Boys (n= 285) Cluster 1 – non-academically-inclined (n= 75, 26.3%) Cluster 2 – academically-inclined (n= 108, 37.9%) Cluster 3 – techno actives (n= 102, 35.8%) Girls (n= 482)  Cluster 1 – academically-inclined (n= 134, 27.8%)  Cluster 2 – active socialisers (n= 276, 57.3%) Cluster 3 – inactive and non-academically-inclined (n= 72, 14.9%) | Boys  Cluster 1 - High PA High/Low SB Cluster 2- High PA High/Low SB  Cluster 3 - High PA High SB  Girls  Cluster 1 - Low PA High/Low SB  Cluster 2 - High PA High SB  Cluster 3 - Low PA Low SB |
| Wang (2011) | Cluster 1 (n= 134, 15.8%) Cluster 2 (n= 107, 12.6%)  Cluster 3 (n= 122, 14.5%) Cluster 4 (n= 386, 45.6%) Cluster 5 (n= 98, 11.5%) | Cluster 1 - High/Low SB Cluster 2 - Low PA Low SB Cluster 3 - High PA High SB Cluster 4 - Low PA High/Low SB Cluster 5 - High PA High SB |
| Note. PA: Physical activity. SB: Sedentary behavior. | | |
